# Dietary interventions in cancer: a systematic review of all randomized controlled trials

**DOI:** 10.1093/jnci/djae051

**Published:** 2024-03-01

**Authors:** Nosakhare Paul Ilerhunmwuwa, Abul Hasan Shadali Abdul Khader, Calvin Smith, Edward R Scheffer Cliff, Christopher M Booth, Evevanne Hottel, Muhammad Aziz, Wade Lee-Smith, Aaron Goodman, Rajshekhar Chakraborty, Ghulam Rehman Mohyuddin

**Affiliations:** One Brooklyn Health/Brookdale University Hospital and Medical Center, Brooklyn, NY, USA; Government Kilpauk Medical College, Chennai, Tamil Nadu, India; Frank H. Netter MD School of Medicine, Quinnipiac University, North Haven, CT, USA; Program on Regulation, Therapeutics and Law, Division of Pharmacoepidemiology and Pharmacoeconomics, Brigham and Women’s Hospital, Harvard Medical School, Boston, MA, USA; Division of Cancer Care and Epidemiology, Queen’s Cancer Research Institute, Kingston, ON, Canada; Division of Hematology, Huntsman Cancer Institute, University of Utah, Salt Lake City, UT, USA; Division of Gastroenterology and Hepatology, University of Toledo, Toledo, OH, USA; Mulford Health Science Library, University of Toledo, Toledo, OH, USA; Division of Blood and Marrow Transplantation, University of California San Diego, San Diego, CA, USA; Division of Hematology, Columbia University Cancer Center, New York, NY, USA; Division of Hematology, Huntsman Cancer Institute, University of Utah, Salt Lake City, UT, USA

## Abstract

**Background:**

Prior systematic reviews addressing the impact of diet on cancer outcomes have focused on specific dietary interventions. In this systematic review, we assessed all randomized controlled trials (RCTs) investigating dietary interventions for cancer patients, examining the range of interventions, endpoints, patient populations, and results.

**Methods:**

This systematic review identified all RCTs conducted before January 2023 testing dietary interventions in patients with cancer. Assessed outcomes included quality of life, functional outcomes, clinical cancer measurements (eg, progression-free survival, response rates), overall survival, and translational endpoints (eg, inflammatory markers).

**Results:**

In total, 252 RCTs were identified involving 31 067 patients. The median sample size was 71 (interquartile range 41 to 118), and 80 (32%) studies had a sample size greater than 100. Most trials (n = 184, 73%) were conducted in the adjuvant setting. Weight or body composition and translational endpoints were the most common primary endpoints (n = 64, 25%; n = 52, 21%, respectively). Direct cancer measurements and overall survival were primary endpoints in 20 (8%) and 7 (3%) studies, respectively. Eight trials with a primary endpoint of cancer measurement (40%) met their endpoint. Large trials in colon (n = 1429), breast (n = 3088), and prostate cancer (n = 478) each showed no effect of dietary interventions on endpoints measuring cancer.

**Conclusion:**

Most RCTs of dietary interventions in cancer are small and measure nonclinical endpoints. Although only a small number of large RCTs have been conducted to date, these trials have not shown an improvement in cancer outcomes. Currently, there is limited evidence to support dietary interventions as a therapeutic tool in cancer care.

Patients with cancer are often interested in exploring different dietary interventions before, during, or after standard cancer treatment. Although a balanced diet clearly contributes to overall health, whether specific dietary interventions can alter the trajectory of cancer or have anticancer activity remains uncertain (1).

Observational studies and nonrandomized trials have frequently reported improved cancer outcomes with adherence to various specific diets (2,3). However, these studies are biased by numerous confounders, both measured and unmeasured. Fundamentally, patients who can follow a specific diet may differ from those who are unable to do so, whether in terms of socioeconomic status, health literacy, pre-existing health, or other factors (4). Approximately 50% of patients with cancer use complementary or alternative medical products (5), and 37% of cancer survivors report using untested dietary interventions (6). There is, therefore, a need to appraise the evidence of the impact of dietary interventions on cancer outcomes.

To our knowledge, no systematic review has comprehensively examined all randomized trials of dietary interventions and supplements in cancer. This systematic review aims to assess all randomized controlled trials (RCTs) of dietary interventions in cancer, including the interventions studied, endpoints measured, patient populations enrolled, and the results of these interventions.

## Methods

### Protocol and registration

This systematic scoping review was performed according to the recommendations of the Preferred Reporting Items for Systematic Reviews and Meta-Analyses (PRISMA) Extension for Scoping Reviews guidelines (7). As no direct patient information was obtained, and the data were gathered from publicly available and deidentified sources, this study was considered exempt from institutional review board review. This was not registered on PROSPERO, as PROSPERO does not permit registration of broad scoping reviews. This study received no external funding.

### Eligibility criteria

RCTs met our inclusion criteria if they focused on dietary intervention, including counseling or the use of dietary supplements in patients with cancer. We included trials on patients with an established history of cancer, including secondary prevention trials in such patients. Trials that focused on patients without cancer, including primary prevention studies, were excluded.

### Publication type and study design

We included only RCTs published as papers in peer-reviewed scientific journals and those published as abstracts in conference proceedings. All other study types were excluded.

### Information sources and search strategy

Our search strategy was restricted to RCTs assessing any dietary intervention published in paper or abstract form from October 1977 to January 2023. The search was last updated on January 20, 2023. A search strategy was made using keywords, and we searched across four electronic databases (MEDLINE, Embase, Web of Science Core Collection, and Cochrane Central Register of Controlled Trials) with the help of an experienced health sciences librarian (W.L.-S). We did not apply any restrictions on language. The Appendix highlights the Embase search strategy. All results were exported to EndNote (v. 20, Clarivate), and duplicate items were removed by successive iterations of EndNote’s duplicate detection features and manual inspection.

### Study selection and screening

Three reviewers (N.P.I., A.H.S., C.S.) worked in pairs in the initial screening of abstracts and titles and assessed eligibility with the full text of selected articles when available. Abstracts from conference proceedings captured on these databases via our search strategy, such as those on Embase, were included. A fourth reviewer (G.R.M.) was consulted to resolve discrepancies and facilitate consensus.

### Data charting and extraction

The independent data charting was done by three reviewers (N.P.I., A.H.S., C.S.). A data charting form was developed and independently piloted by a random sample of 10% of the included articles. It was modified as required based on feedback from within the team. The following characteristics of studies were identified: median age of participants, sample size, trial setting, primary endpoint and its outcome, country origin of trial, cancer types being studied, trial phase, blinding, and the dietary interventions employed. After initial charting, the reviewers validated the accuracy of data points and reached a consensus on each input. When discrepancies or uncertainties arose among reviewers, a fourth reviewer (G.R.M.) was consulted to facilitate consensus, and a dietician (E.H.) provided input.

### Study outcomes

We defined standard quality-of-life primary endpoints as those using validated quality-of-life (QoL) measurements such as the European Organization for Research and Treatment of Cancer Quality of Life Questionnaire-Core 30 (EORTC QLQ-C30) (8), Functional Assessment of Chronic Illness Therapy-Fatigue (FACIT-F) (9), Functional Assessment of Chronic Illness Therapy—General (FACIT-G) (9), and Short Form 36 (SF-36) (10) health questionnaires. We defined nonstandard quality-of-life primary endpoints as endpoints that measured symptom burden without the use of standardized scales, such as chemotherapy or radiation-induced adverse events, infection rates, unspecified measurement scales of QoL, and functional outcomes (such as fatigue measured without the use of a standardized scale). Cancer measurement endpoints included response rates, clinical or biochemical recurrences, progression-free survival, relapse-free survival, time until cancer recurrence, and overall survival. Endpoints that focused on a related pre-cancer, such as polyps or actinic keratosis, were considered a cancer measurement endpoint. Perioperative endpoints included postoperative length of stay, complications (infective and noninfective), and functional outcomes (fatigue or pain) in the perioperative setting. Blinding categorization was defined as follows: single-blinded studies were studies in which participants were blinded to treatment assignment. Double-blinded were defined as studies in which both health-care providers and patients were masked to treatment assignment, and triple-blinded trials also blinded researchers to treatment assignment. Studies that did not report blinding for study participants or investigators were deemed open label. We analyzed countries where trials were conducted using the World Bank data to classify countries according to their Gross National Income (GNI) per capita, using it as a reference standard (11).

### Trial setting

To explore how dietary interventions are ascertained across the cancer treatment continuum, we categorized trials into four discrete groups to capture the setting in which they were studied. The “adjuvant” setting included interventions applied concurrently with curative-intent chemotherapy, radiotherapy, surgical resection, hematopoietic stem cell transplantation, or other procedures. All perioperative interventions were classified in this category. The “palliative” setting included interventions administered in patients who were being treated with palliative intent. This included patients with metastatic cancer, regardless of whether they were receiving chemotherapy. The third category was secondary prevention and/or survivorship among patients who have completed curative intent treatment and have no evidence of cancer. A fourth category was trials specifically done for patients with either cancer cachexia or malnutrition. Trials that fit none of these categories or included patients in multiple settings were classified as miscellaneous.

## Results

**Figure 1.**
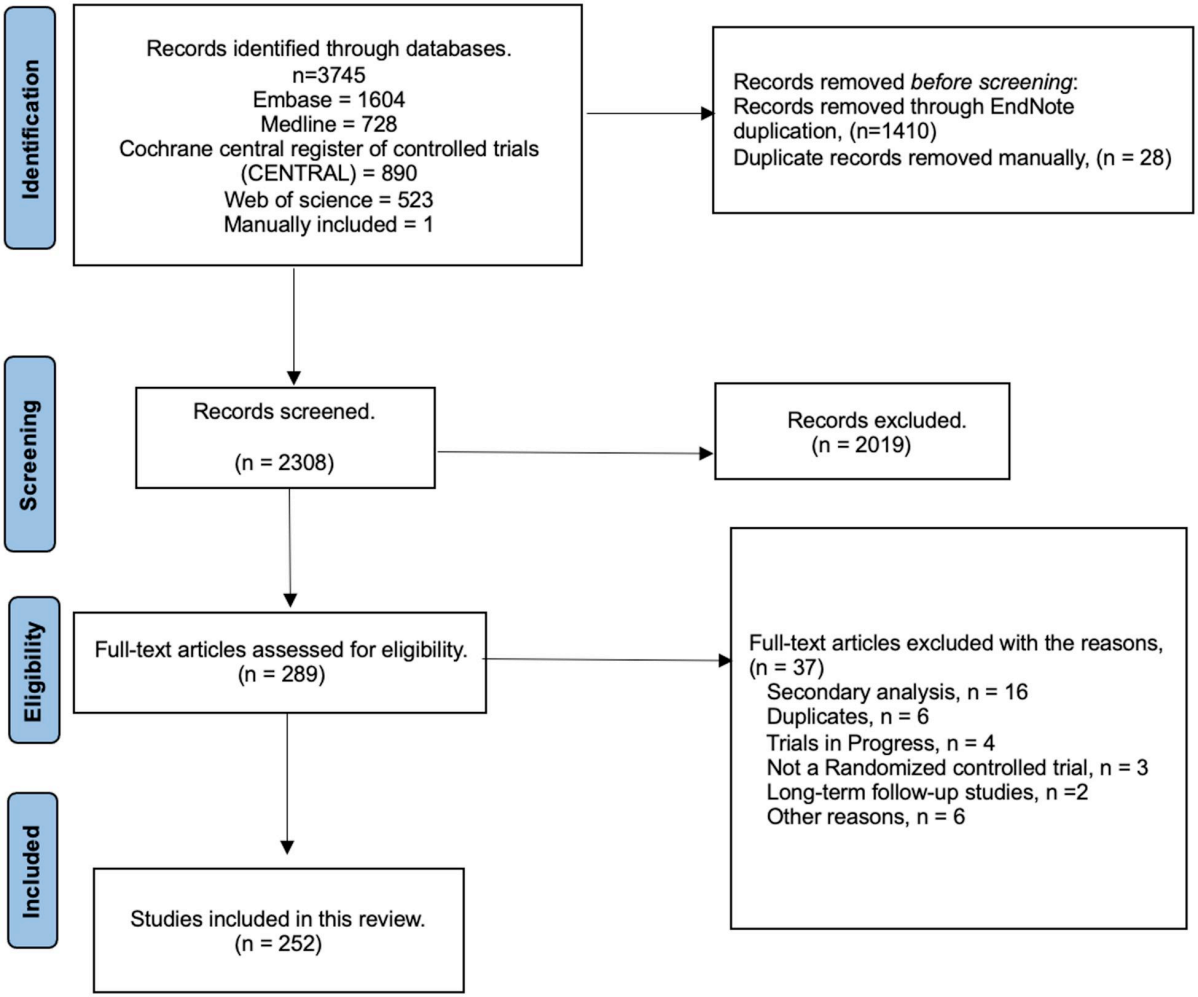
Preferred reporting items for systematic reviews and meta-analyses (PRISMA) flow diagram.

The initial search strategy identified 3745 studies, of which 252 were RCTs (Supplementary Table 1, available online; lists characteristics of all studies). Figure 1 highlights our screening process. The median sample size of eligible RCTs was 71 patients (Interquartile range: 41-118). Eighty studies (32%) had a sample size greater than 100. Characteristics of included studies are listed in Table 1.

**Table 1.** Characteristics of included studies

Median sample size		71 (Interquartile range 41, 118)
Median age		61 (5,77)
Studies by countries stratified by income (N = 252)	High-income Countries	162 (64.3%)
	Upper-middle-Income Countries	77 (30.5%)
	Lower-middle-Income Countries	13 (5.2%)
	Low-income Countries	None
Studies grouped by primary endpoint	Weight, body composition, or muscle	64 (25.4%)
	Translational or preclinical endpoints	52 (20.6%)
	Nonstandard measurements of QoL	38 (15.1%)
	Postoperative endpoints	35 (13.9%)
	Compliance or adherence or feasibility	21 (8.3%)
	Direct measurements of cancer	20 (7.9%)
	Quality-of-life	15 (6.0%)
	Overall survival	7 (2.8%)
Cancers studied (N = 252), %	Gastrointestinal cancers	97 (38.5%)
	Mixed cancers	36 (14.3%)
	Breast cancer	28 (11.1%)
	Prostate cancer	29 (11.5%)
	Head and neck cancer	25 (9.9%)
	Hematologic malignancies	12 (4.7%)
	Gynecologic malignancies	9 (3.6%)
	Skin cancers	4 (1.6%)
	Cancer type not specified	3 (1.2%)
	Other^a^	9 (3.6%)
Trial setting (N = 252), %	Adjuvant	184 (73.0%)
	Miscellaneous or Mixed	26 (10.3%)
	Secondary Prevention and/or Survivorship	21 (8.3%)
	Palliative	14 (5.5%)
	Cachexia or Malnutrition	7 (2.8%)

a Pediatric malignancies (n = 2, 0.8%), lung cancer (n = 2, 0.8%), malignant gliomas (n = 2, 0.8%), bladder (n = 1, 0.4%), renal (n = 1, 0.4%), thyroid (n = 1, 0.4%).

### Cancer populations

The most common cancers studied were gastrointestinal (n = 97, 38%), followed by mixed cancer populations (n = 36, 14%), breast cancer (n = 28, 11%), prostate cancer (n = 29, 12%), head and neck cancer (n = 25, 10%), hematological malignancies (n = 12, 5%), and gynecological malignancies (n = 9, 4%).

### Dietary interventions

The types of dietary interventions are listed in Supplementary Table 2 (available online). Diets were categorized into 7 categories, which included nutrient-modified diets (eg, supplemented vitamins, minerals, amino acids, or certain foods), nutritional counseling, energy-modified diets (these diets modified caloric intake or relative proportions of macronutrients), nutrition support (eg, parenteral and enteral nutrition), restrictive eating patterns (eg, ketogenic diet, neutropenic diet, vegan diet, plant-based diet, Chinese medicated diet), the Mediterranean diet, and fiber-modified diets. The most common intervention was nutrient-modified diets (n = 126, 50%), followed by nutrition counseling interventions (n = 44, 17%), energy-modified diets (n = 34, 13%), nutrition support (n = 27, 11%), restrictive eating patterns (n = 11, 4%), Mediterranean diets (n = 6, 2%), and fiber-modified diets (n = 4, 2%).

### Settings and endpoints

**Figure 2.**
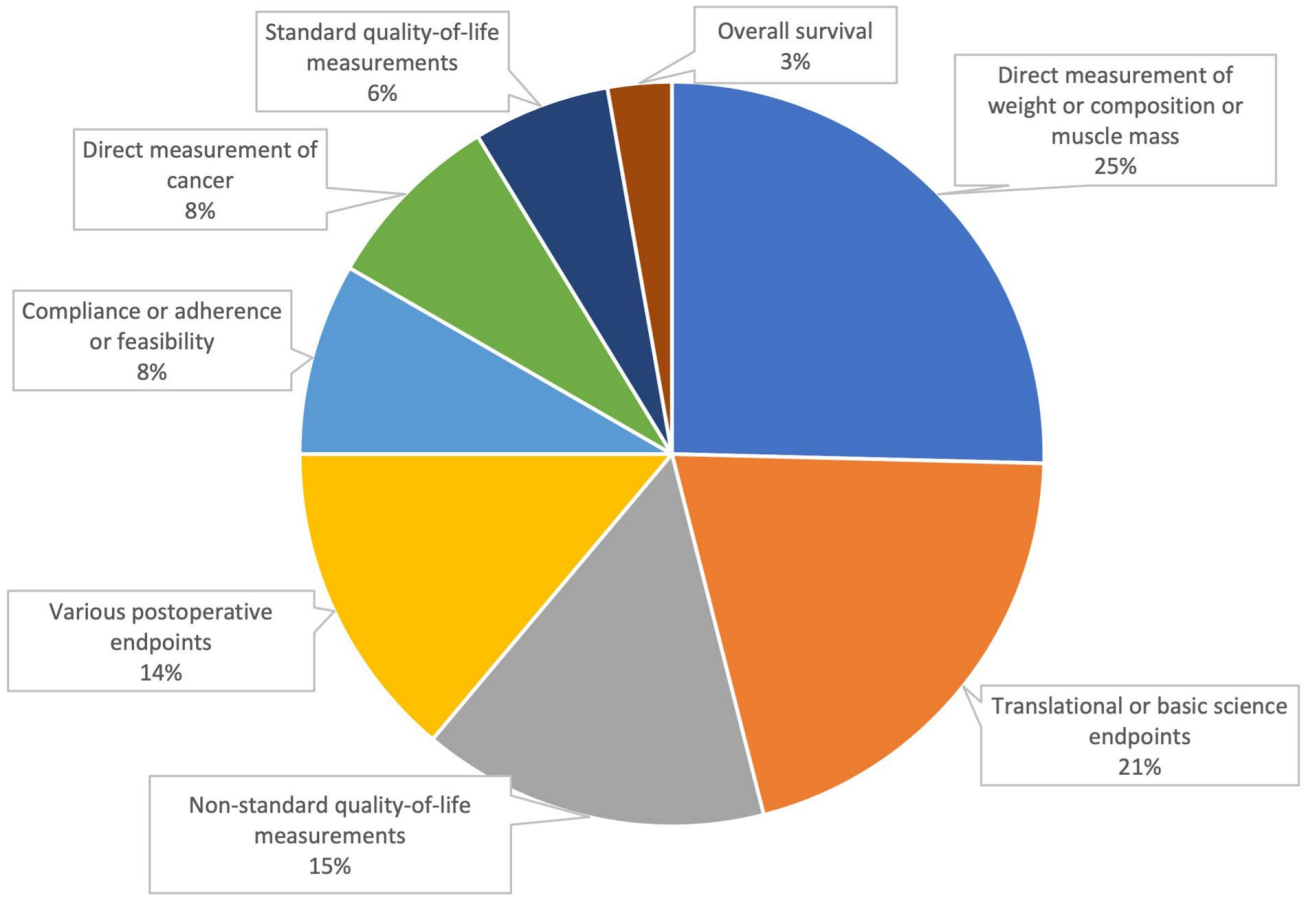
Primary endpoints of dietary intervention trials.

**Figure 3.**
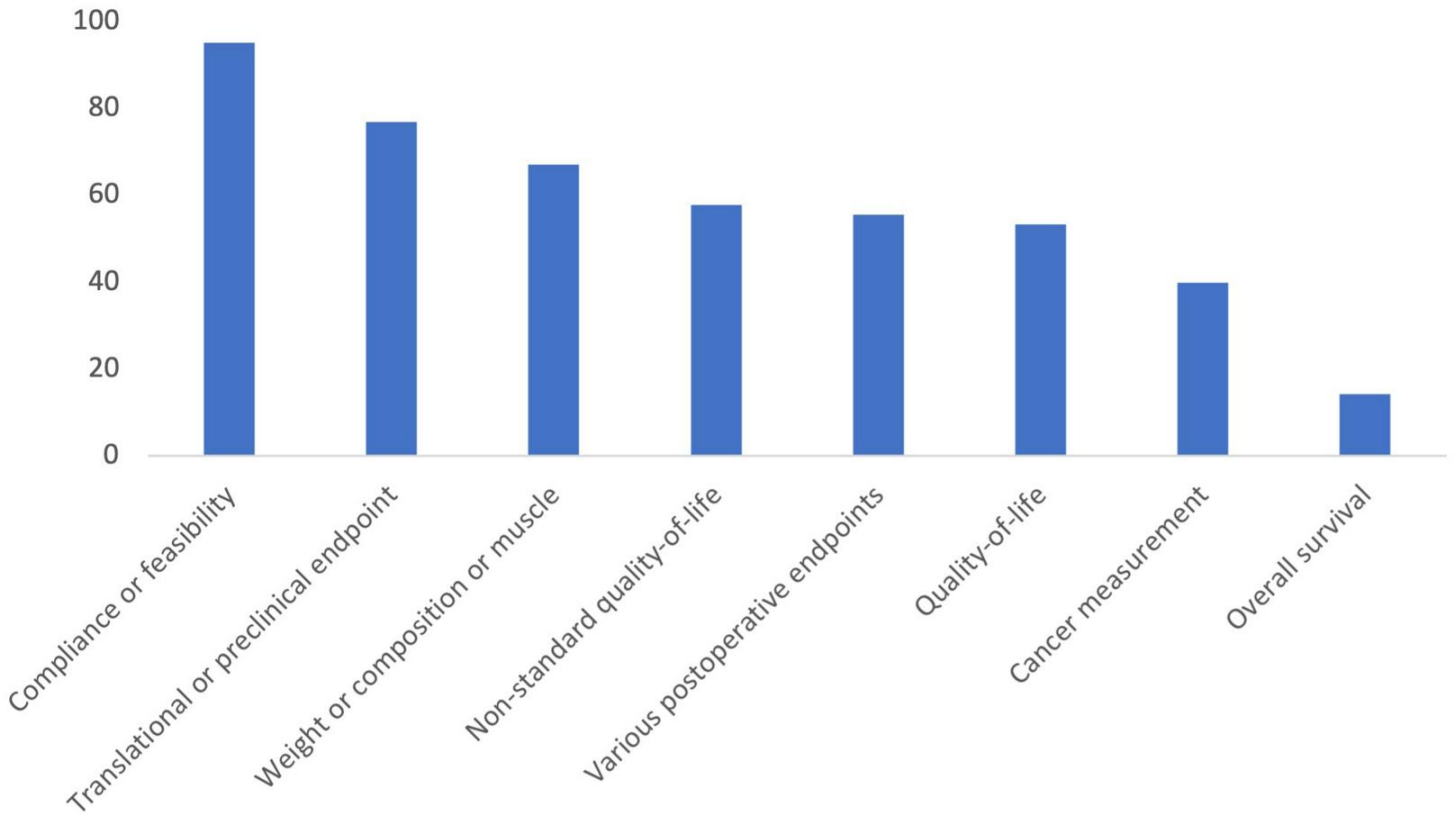
Percentage of RCTs with achieved primary endpoint by endpoint categorization. Endpoints from RCTs testing dietary interventions in cancer patients were categorized into the following categories: Compliance or feasibility, Translational or preclinical, measurements of Weight or composition or muscle mass, Nonstandard quality-of-life, Various postoperative endpoints, Quality-of-life, Cancer measurements, and Overall survival. Quality-of-life endpoints were defined as validated measurements such as the EORTC QLQ-C30, FACIT-F, FACIT-G, and SF-36 health questionnaires. Nonstandard quality-of-life were measurements of symptom burden without the use of standardized scales, such as chemotherapy or radiation-induced adverse events, infection rates, unspecified measurement scales of QoL, and functional outcomes (such as fatigue). Cancer measurement endpoints were defined as response rates, clinical/biochemical recurrences, progression-free survival, relapse-free survival, and time until cancer recurrence.

Endpoints used in included trials are summarized in Figures 2 and 3, and demonstrate the proportions of trials meeting their primary endpoint based on the type of endpoint used. Most trials (n = 184, 73%) were conducted in the adjuvant setting. Weight or body composition endpoints and translational endpoints were the most common primary endpoints (n = 64, 25%; n = 52, 21%, respectively). Direct cancer measurements were a primary endpoint in 20 (8%) studies, and overall survival was the primary endpoint in 7 (3%) studies.

### Trials that measured cancer outcomes and overall survival

Among the 20 studies with a primary endpoint of cancer (or precancer) measurement, 8 (40%) studies achieved their endpoint (Table 2). Among the 20 studies for which direct measurement of cancer or associated precancer was a primary endpoint, the endpoints were biochemical recurrences of prostate-specific antigen in 8 (40%) studies, clinical recurrence of cancer in 3 (15%) trials, progression-free survival in 3 (15%) studies, response rates in 2 (10%) studies, relapse-free survival in 1 (5%), disease-free survival in 1 (5%), and occurrence of new skin cancers and precancerous keratosis in 1 (5%) trial.

**Table 2.** Details of studies with a primary endpoint of cancer measurement or overall survival^a^

Study	Sample size	Dietary intervention	Trial setting	Primary endpoint	Endpoint met?
Dequadros Camargo et al. (14)	30	Fish oil supplements	Adjuvant	Time to disease progression (days)	Yes
Khodabakhshi et al. (15)	80	Ketogenic diet	Adjuvant	Response rate (reduction in tumor size)	Yes
Nojiri, S. et al. (16)	51	Branched-chain amino acids supplements	Adjuvant	Time until intrahepatic recurrence of HCC	Yes
Rangel Huerta et al. (18)	74	Lycopene supplements	Adjuvant	PSA levels	Yes
Thomas et al. (47)	203	Supplements with pomegranate seeds, green tea, broccoli, and turmeric	Adjuvant	Median rise in PSA	Yes
De Waele et al. (32)	60	Intensive personalized dietary counseling	Adjuvant	Overall Survival	Yes
Black et al. (12)	115	Low-fat diet	Secondary prevention or survivorship	Cumulative incidence of new AK	Yes
Chen et al. (13)	386	500 mg of nicotinamide twice daily	Secondary prevention or survivorship	Number of new histologically confirmed nonmelanoma skin cancers	Yes
Kranse et al. (17)	37	Supplements with plant estrogens, carotenoids, and selenium	Misc or mixed	Slope of the rise in PSA	Yes
Bougnoux et al. (23)	65	Long-chain	Adjuvant	Progression-free survival	No
		PUFA			
		supplements			
Voss et al. (31)	50	Ketogenic diet	Adjuvant	Progression-free survival rate at six months	No
Bourdel-Marchasson et al. (35)	341	Dietary counseling aiming to increase energy and protein intake	Adjuvant	One-year survival	No
Lin et al. (35)	97	Zinc supplements	Adjuvant	Overall survival	No
Sykorova et al. (37)	44	Glutamine rich parenteral nutrition	Adjuvant	Overall survival	No
Toma et al. (38)	214	Beta-carotene supplements	Adjuvant	Overall survival	No
Freedland et al. (25)	57	Low-carbohydrate diet	Palliative	PSA doubling time	No
Van Zweeden et al. (30)	82	Vitamin B_12_ and B_9_ supplements	Palliative	Response rate	No
Antunac et al. (33)	71	Vitamin D supplements	Palliative	Overall survival	No
Baldwin et al. (34)	358	Nutritional supplements with high-calorie diet	Palliative	One-year survival	No
Alberts et al. (21)	1429	High-fiber diet	Secondary prevention or survivorship	Presence or absence of new adenomas at the time of follow-up colonoscopy	No
Bosland et al. (22)	177	Soy protein supplements	Secondary prevention or survivorship	Biochemical recurrence of prostate cancer (PSA levels)	No
DeVere White et al. (24)	66	High-dose isoflavone supplements	Secondary prevention or survivorship	Serum PSA	No
Chlebowski et al. (20)	2437	Low-fat diet	Secondary prevention or survivorship	Relapse-free survival	No
Johansson et al. (28)	104	Vitamin D supplements	Secondary prevention or survivorship	Disease-free survival	No
Parsons et al. (26)	478	Telephone-based counseling	Secondary prevention or survivorship	Time to progression (PSA)	No
Pierce et al. (29)	3088	Diet rich in vegetables, fruits, and fiber, but with low fat	Secondary prevention or survivorship	Invasive breast cancer event (recurrence or new primary) and all-cause mortality	No
Schröder et al. (27)	49	Soy-based supplements	Misc or mixed	PSA slope and doubling time	No

a AK = actinic keratosis; HCC = hepatocellular cancer; PSA = prostate specific antigen.

The 8 studies that met their endpoint evaluating cancer-related outcomes consisted of a low-fat diet study at reducing actinic keratosis in patients with skin cancer (n = 115) (12), a trial supplementing nicotinamide aimed at reducing new histologically confirmed nonmelanoma skin cancers in patients with a history of nonmelanoma skin cancer (n = 386) (13), a fish oil study that aimed at reducing time to progression for gastrointestinal (GI) cancers (n = 30) (14), a study of a ketogenic diet that aimed at increasing breast cancer response rates (n = 80) (15), a branched-chain amino-acid supplementation study measuring the recurrence of hepatocellular cancer (n = 51) (16), and three trials of various supplements or diets that aimed at reducing prostate specific antigen (PSA) values (n = 37, 74, and 203, respectively) (17-19).

The 12 studies that did not meet their endpoint when evaluating cancer-related outcomes included a low-fat diet in breast cancer (n = 2437) on relapse-free survival (20), a high-fiber diet study on adenoma rates among patients with colorectal cancer (n = 1429) (21), a soy-protein supplement study on prostate cancer recurrence rates (n = 177) (22), a long-chain fatty acid study on breast cancer progression-free survival (n = 65) (23), high-dose isoflavone supplementation, low-carbohydrate diet, telephone-based dietary counseling on PSA rates (n = 66 and 57, respectively) (24,25), a telephone-based dietary counseling study measuring time to progression (n = 478) (26), a soy-based dietary supplementation study on PSA doubling time (n = 49) (27), a vitamin D supplementation study on skin cancer (n = 104) (28), a diet high in vegetables, fruits, and fiber study on breast cancer survival (n = 3088) (29), a vitamin B_12_ and folic acid study on response rates in GI cancers (n = 82) (30), and an intermittent fasting and ketogenic diet study on progression-free survival of gliomas (n = 50) (31).

One trial with a primary endpoint of overall survival met its endpoint (n = 60) (32). This trial tested counseling patients to increase caloric intake among a mixed cancer population and showed that at 12 months follow-up, the control group had a median survival of 45.5 weeks vs not reached in the intervention group (*P* = .0378) (32).

Trials that did not meet their overall survival endpoint included a vitamin D supplement study in colorectal cancer (n = 71) (33), two studies that focused on increasing caloric intake with one among patients with GI cancers, non-small-cell lung cancers, and mesothelioma (n = 358) (34) and the other in a mixed cancer population (n = 341) (35), a zinc supplementation trial for patients with head and neck cancer (n = 97) (36), a trial that tested a glutamine-enriched parental supplementation in patients with hematological malignancies (n = 44) (37), and a study that used beta-carotene supplements for head and neck cancer patients (n = 214) (38).

### Likelihood of meeting endpoint based on trial design

The majority of trials reported either an open-label design or did not report any blinding procedures, 184 (73%). A total of 19 studies (8%) incorporated a single-blind design, and 49 (19%) studies reported a double- or triple-blind design. Trials that were open label were more likely to meet their primary endpoint (68%) vs those that were blinded (38%), χ^2^ (1, n = 252) equals 18.9, *P* less than .05.

A total of 79 (31%) studies used counseling techniques, whereas the remaining trials (n = 173, 68.6%) directly administered dietary meals or nutritional supplements. In total, 56 (70.9%) of the studies using counseling and 105 (60.7%) of studies providing meals or supplementation met their primary endpoint, χ^2^ (1, n = 252) equals 2.44, *P* equals .12.

## Discussion

This is the first comprehensive systematic review to assess all RCTs evaluating dietary interventions in patients with cancer. We find that dietary intervention trials for cancer patients are typically small and most often assess translational and body composition endpoints. These trials often met their primary endpoints, showing improvements in translational parameters and body composition with dietary interventions. In addition, most trials were done in the adjuvant setting in conjunction with other approaches such as chemotherapy, radiation, and surgery. However, direct measurements of cancer were a primary endpoint in only 8% of studies.

Only a small number of trials both had adequate statistical power and assessed an endpoint of cancer measurement; these generally did not meet their endpoint, such as large trials in colon cancer (n = 1429) (21), and breast cancer (n = 3088) (29). Our results cumulatively indicate that it has been difficult to demonstrate the clinical benefit of specific dietary interventions as a therapeutic anticancer strategy in RCTs in patients with cancer. Given the lack of adequately powered RCTs with clinical endpoints, the benefits of dietary interventions remain largely untested. However, based on the existing evidence from the small number of conducted large RCTs, there is currently very limited evidence to support dietary interventions as a therapeutic tool in cancer care. These results serve as an important call for further high-quality large RCTs in this space, powered to show improvements in clinically meaningful endpoints.

Our study sought to systematically evaluate the collective RCT evidence of diverse dietary interventions in cancer. Prior reports have focused on specific dietary patterns across multiple cancer populations, some comprising RCTs and observational studies (39-44). These systematic reviews have observed that dietary interventions for cancer patients or survivors are feasible with positive outcomes for translational endpoints (41,44,45). Our work corroborates this finding, indicating that many feasibility and translational trials meet their primary endpoints; it seems, however, that many of these interventions are not subsequently tested in larger trials.

Some RCTs testing dietary interventions and measuring cancer outcomes met their primary endpoint, but these trials have been limited by small sample sizes and lack of reproducibility. In prostate cancer, although some trials of dietary interventions powered for rise of PSA met their endpoint (17,18,19), other trials of dietary interventions or counseling with longer follow-up did not, including a large study of 478 patients in which dietary interventions led to an increase in vegetable consumption but did not change time to progression (26). In other tumor types, trials that met their endpoints had small sample sizes necessitating confirmation with larger trials. For example, a fish oil study (n = 30) reduced time to progression for GI cancers (593 days vs 330 days) (14), and a branched-chain amino-acid supplementation trial (n = 51) also reduced recurrence (44% vs 68% at 3 years) of hepatocellular cancer (16). Additionally, one trial that showed improved overall survival with counseling to increase caloric intake had a sample size of 60 patients and a variety of cancers were included, limiting its applicability to routine clinical practice in terms of recommending a specific diet for specific patients with cancer (32). An exception to this is an oral nicotinamide trial with a sample size of 386 participants, which was effective in reducing new nonmelanoma skin cancers in those who already had skin cancers, with a distinct biological mechanism explaining this effect (13). Overall, although these trials have demonstrated that some dietary interventions may have an effect on cancer, due to the limitations of these studies, there is insufficient evidence to recommend specific diets (beyond general lifestyle changes for better health) for patients with cancer. Future work should look to replicate positive findings seen in small studies in trials with larger sample sizes.

Several RCTs with adequate statistical power have been conducted to evaluate the impact of interventions on cancer outcomes, particularly in breast and prostate cancers. Unfortunately, the results of these trials have often been negative. For example, a large RCT that used a telephone-based counseling method to increase vegetable intake in patients with prostate cancer showed no difference in PSA between the control and intervention arms (n = 478) (26). Additionally, a well-designed trial to increase fruit and vegetable intake among breast cancer patients showed no change in breast cancer recurrence (n = 3088) (29). Although an RCT showed a potential effect of a low-fat diet in breast cancer (n = 2437) with improved relapse-free survival (relapse events in 9.8% of the intervention group vs 12.4% in the control group) (20), this trial did not have a statistically significant *P* value for the primary endpoint when analyzed via the prespecified stratified log-rank test (*P* = .077), indicating that the results could be due to chance alone. When considering these results, RCTs cumulatively indicate that dietary interventions studied to date generally have offered limited benefits in reducing the burden of cancer. In these RCTs, the compliance to the intervention was frequently sufficient to lead to a change in the diet as measured by surveys and nutritional indices (such as plasma carotenoids), but this change did not translate to a difference in cancer outcomes such as progression-free survival. This challenges the previously suggested causal connections between adherence to specific diets seen in observational data and cancer outcomes, highlighting the role confounding may play in observational studies that suggest a large effect size of diet in cancer (2,3).

We found that smaller trials assessing translational endpoints or endpoints directly measuring weight or body composition often achieved their primary endpoint (77% and 67%, respectively), whereas larger RCTs are often negative. This shows intuitively that dietary interventions may help maintain or restore an ideal body weight or improve certain laboratory values, as they are known to do in patients without cancer. Nevertheless, these studies provide limited information about the potential clinical benefit of dietary interventions as therapeutic measures to reduce cancer burden.

Certain diets such as the ketogenic diet, or other diets that aim to starve cancer cells of sugar, attract considerable media attention as a mechanism to cure cancer (46). Our review finds no convincing evidence that any diet “cures” cancer, with no large robust randomized study demonstrating this, and smaller studies producing conflicting results (15,46).

The strength of this systematic review lies in its focus on RCTs that have investigated the role of diet in patients with cancer. The review also encompasses studies from diverse geographical regions and various cancer types, lending weight to the generalizability of our conclusions. A limitation is that some of the included abstracts did not clearly describe the study’s primary endpoint, requiring us to infer the primary endpoint. In addition, because no RCTs were performed in low-income countries and only 5% of RCTs were performed in lower-middle-income countries (Table 1), these results may not be generalizable to patients in countries where nutritional deficiencies may be more prevalent.

Additionally, interpreting RCTs testing dietary interventions in cancer may be subject to limitations. These include insufficient blinding leading to an increased probability of a false negative finding due to contamination of the control arm, and self-selected enrollment of comparatively more motivated patients, which may decrease generalizability to a broader population. Furthermore, dietary interventions can be very heterogenous (as can cancer), and results of dietary interventions in one cancer may not extrapolate to others. A final limitation of our analysis is that no formal meta-analytic method was employed due to the heterogeneity of assessed interventions and outcomes.

Our review confirms that dietary interventions in these studies often positively impact translational endpoints, symptom burden, and/or body composition. However, despite smaller RCTs often meeting their primary outcome, when larger RCTs of dietary interventions directly measuring cancer are conducted, they have not yet consistently shown any effect on cancer progression. Given immense interest from patients in this field, it is paramount to generate high-quality, randomized data with clinical endpoints, as the results of observational studies are subject to confounding and cannot be relied on. Although there are challenges in running RCTs in this space, they remain essential to further our understanding of diet as an anticancer modality. Our results suggest that larger studies directly measuring cancer should be prioritized for funding rather than further small studies with nonclinical endpoints. An ideal randomized trial in this setting would be a trial of a dietary intervention that is scalable and easy to adhere to, with adequate power to capture a meaningful difference in a clinically relevant endpoint (eg, cancer response rate, progression-free survival, or validated quality of life measurement). Such a trial may be done in conjunction with effective cancer pharmacological therapy and may be designed to isolate the effect of the dietary intervention. A holistic, multidisciplinary approach to optimizing the health of patients with cancer remains paramount; however, there is currently limited evidence to support specific dietary interventions as a therapeutic tool to target cancer.

## Supplementary Material

djae051_Supplementary_Data

## Data Availability

All data used to inform this paper are publicly available.
