# Supplementary material for: Dietary interventions in cancer: a systematic review of all randomized controlled trials
Source: J Natl Cancer Inst. 2024 Mar 1;116(7):1026–34. doi: 10.1093/jnci/djae051 (PMC11223872; doi:10.1093/jnci/djae051)
Supplement: djae051_Supplementary_Data [file djae051_supplementary_data.pdf]

Supplementary Table 1

Here is a listing of all included RCTs testing dietary interventions for cancer patients with reported sample size and primary endpoint as well as the assigned endpoint categorization and trial setting.

Title	Sample size	Primary Endpoint	Endpoint Categorized	Trial Setting	PubMed ID
Lack of effect of a high-fiber cereal supplement on the recurrence of colorectal adenomas	1429	Presence or absence of new adenomas	Cancer measurement	Secondary prevention/survivorship	10770890
Impact of Preoperative Acetaminophen and Carbohydrate Loading on Pain and Functional Status in Patients Undergoing Mohs Micrographic Surgery for Nonmelanoma Skin Cancers	96	Perioperative and radiation pain, thirst, hunger, anxiety, and fatigue	Postoperative Misc endpoint	Adjuvant	31725692
Oral nutritional supplement (ONS) reduced weight loss and optimised preoperative energy and nutrient intake in colorectal cancer patients undergoing surgery	30	Weight loss and preoperative energy intake	Weight/Composition/Muscle	Adjuvant	Not found
A Translational Randomized Trial of Perioperative Arginine Immunonutrition on Natural Killer Cell Function in Colorectal Cancer Surgery Patients	24	NK cell cytotoxicity, activity, and cell number	Translational/Basic Science Endpoint	Adjuvant	35913670
Vitamin D Supplementation and Survival in Metastatic Colorectal Cancer	71	Overall survival	Overall Survival	Palliative	29533115
Effects of perioperative Eicosapentaenoic acid-enriched oral nutritional supplement on lean body mass after total gastrectomy for gastric cancer	126	Lean body mass	Weight/Composition/Muscle	Adjuvant	30854113
Efficacy of ω-3 supplementation on nutritional status, skeletal muscle, and chemoradiotherapy toxicity in cervical cancer patients: A randomized, triple-blind, clinical trial conducted in a middle-income country	46	Skeletal muscle quality and quantity, nutritional status, and chemotherapy toxicity	Weight/Composition/Muscle	Adjuvant	Not found
Effects of a parallel-arm randomized controlled weight loss pilot study on biological and psychosocial parameters of overweight and obese breast cancer survivors	21	Body weight, biomarkers, QOL, and fitness level	Weight/Composition/Muscle	Secondary prevention/survivorship	28702218
Phase II prospective randomized trial of a low-fat diet with fish oil supplementation in men undergoing radical prostatectomy	55	IGF1 Levels, serum IGFBP-1, markers of proliferation, and apoptosis	Translational/Basic Science Endpoint	Adjuvant	22027686
Randomized study to compare the compliance of two prescription of nutritional supplementation in oncology inpatients	36	Compliance	Complaince/Adherence/Feasibility	Misc/Mixed	Not found
The Impact of a Ketogenic Dietary Intervention on the Quality of Life of Stage II and III Cancer Patients: A Randomized Controlled Trial in the Caribbean	40	Quality of Life (EORTC QLQ-C30)	Quality of Life	Adjuvant	32791011
Mediterranean-style dietary pattern improves cancer-related fatigue and quality of life in men with prostate cancer treated with androgen deprivation therapy: A pilot randomised control trial	23	Cancer related fatigue and quality of life (FACIT-F) and (FACIT-G)	Quality of Life	Misc/Mixed	35565100
The impact of a mediterranean-style dietary pattern on cancer-related fatigue and quality of life in men with prostate cancer treated with androgen deprivation therapy: A pilot study	12	Cancer related fatigue and quality of life [FACT-F] and [FACT-G]	Quality of Life	Misc/Mixed	Not found
Simple nutritional intervention in patients with advanced cancers of the gastrointestinal tract, non-small cell lung cancers or mesothelioma and weight loss receiving chemotherapy: a randomised controlled trial	358	Overall survival	Overall Survival	Palliative	21733143
The effect of perioperative immunonutrition on surgical complications and quality of life in patients with head and neck cancer	34	Nutritional status, anthropometrics and QoL (EORTC QLQ-C30, EORTC QLQ-H & N35)	Postoperative Misc endpoint	Adjuvant	Not found
The effects of short-term fasting on quality of life and tolerance to chemotherapy in patients with breast and ovarian cancer: A randomized cross-over pilot study	50	Quality of life (FACIT), fatigue, and well-being	Quality of Life	Adjuvant	29699509
The use of a protein and energy dense eicosapentaenoic acid containing supplement for malignancy-related weight loss in children	52	Percent subjects showing weight loss and BMI loss	Weight/Composition/Muscle	Adjuvant	19090549
Comparing diets for weight loss and improvement in biomarkers in men with prostate cancer on surveillance: A pilot study	30	Improvement in nutritional biomarkers	Translational/Basic Science Endpoint	Secondary prevention/survivorship	Not found
Influence of dietary factors on actinically-induced skin cancer	115	Occurence of precancerous keratoses and NMSC	Cancer measurement	Secondary prevention/survivorship	9920444
A double-blind phase III trial of immunomodulating nutritional formula during adjuvant chemoradiotherapy in head and neck cancer patients: IMPATOX	180	Incidence of grade 3/4 mucositis	Non-standard Quality of Life	Adjuvant	32936874
Effect of soy protein isolate supplementation on biochemical recurrence of prostate cancer after radical prostatectomy: A randomized trial	177	Biochemical recurrence of cancer (PSA level)	Cancer measurement	Secondary prevention/survivorship	33764851
Modification of the response to chemotherapy of HER2 negative metastatic breast cancer by lipids of marine origin: A controlled, randomized, double blind dietary supplementation trial	65	Progression free survival	Cancer measurement	Adjuvant	Not found
Nutritional advice in older patients at risk of malnutrition during treatment for chemotherapy: a two-year randomized controlled trial	341	One year survival	Overall Survival	Adjuvant	25265392
Glutamine supplementation in cancer patients receiving chemotherapy: A double-blind randomized study	65	Incidence/severity of diarrhea and tumor response	Non-standard Quality of Life	Adjuvant	9263281
Preoperative oral arginine and n-3 fatty acid supplementation improves the immunometabolic host response and outcome after colorectal resection for cancer	200	Immune response, gut oxygenation, and postoperative infection	Postoperative Misc endpoint	Adjuvant	12464864
Immune and nutritional effects of early enteral nutrition after major abdominal operations	60	Immunological and nutritional variables	Translational/Basic Science Endpoint	Adjuvant	8639722

Effect of fish oil on appetite and other symptoms in patients with advanced cancer and anorexia/cachexia: a double-blind, placebo-controlled study	60	Appetite, tiredness, calorie intake, weight loss, nausea, well being, and nutritional status	Non-standard Quality of Life	Palliative	12506181
Whey Protein Supplementation Improves Nutritional Status, Glutathione Levels, and Immune Function in Cancer Patients: A Randomized, Double-Blind Controlled Trial	48	Serum albumin, glutathione (GSH) levels, immunity, and inflammatory markers	Translational/Basic Science Endpoint	Adjuvant	29565716
Monitoring dietary change in a low-fat diet intervention study: advantages of using 24-hour dietary recalls vs food records	290	Evaluation of dietary assessment methods for monitoring change in fat intake	Complaince/Adherence/Feasibility	Misc/Mixed	Not found
The influence of low fat, low lactose diet on diarrhoea during pelvic radiotherapy	143	Radiation-induced diarrhea, nausea, and vomiting	Non-standard Quality of Life	Adjuvant	7900512
The impact of preoperative immunonutrition and other nutrition models on tumor infiltrative lymphocytes in colorectal cancer patients	28	Tumor infiltrative lymphocytes in endoscopic tissue biopsy specimens	Translational/Basic Science Endpoint	Adjuvant	23010614
A Whole-Food, Plant-Based (WFPB) dietary intervention to improve outcomes in patients with metastatic breast cancer	28	Compliance, weight, cardiometabolic outcomes, and cancer progression markers	Complaince/Adherence/Feasibility	Palliative	38045318
A Dietary Intervention for Recurrent Prostate Cancer After Definitive Primary Treatment: Results of a Randomized Pilot Trial	36	Compliance	Complaince/Adherence/Feasibility	Adjuvant	18400281
Effect of Nutritional Supplementation Enriched with Eicosapentaenoic Acid on Inflammatory Profile of Patients With Oral Cavity Cancer in Antineoplastic Pretreatment: A Controlled and Randomized Clinical Trial	64	Inflammatory profile	Translational/Basic Science Endpoint	Adjuvant	28128983
Pancreatic enzyme supplementation after gastrectomy for gastric cancer: a randomized controlled trial	43	Nutritional status and QOL	Weight/Composition/Muscle	Adjuvant	28804801
Social cognitive outcomes are associated with improvements in mobility performance following lifestyle intervention in prostate cancer patients undergoing androgen deprivation therapy	32	Lift and carry performance and mobility-related self-efficacy belief	Non-standard Quality of Life	Adjuvant	35085341
Role of enteral immunonutrition in patients with gastric carcinoma undergoing major surgery	40	Immune and nutritional parameters	Translational/Basic Science Endpoint	Adjuvant	15851366
Effects of Five-Step Nutritional Interventions Conducted by a Multidisciplinary Care Team on Gastroenteric Cancer Patients Undergoing Chemotherapy: A Randomized Clinical Trial	78	Nutritional status via Patient-Generated Subjective Global Assessment	Weight/Composition/Muscle	Adjuvant	35903847
A Phase 3 Randomized Trial of Nicotinamide for Skin-Cancer Chemoprevention	386	Number of new histologically confirmed non-melanoma skin cancers	Cancer measurement	Secondary prevention/survivorship	26488693
The influence of low-carbohydrate diets on the metabolic response to androgen-deprivation therapy in prostate cancer	40	Serum metabolites	Translational/Basic Science Endpoint	Misc/Mixed	33949711
Arginine, glutamine, and fish oil supplementation in cancer patients treated with concurrent chemoradiotherapy: A randomized control study	88	Incidence of acute toxicities	Non-standard Quality of Life	Adjuvant	31146957
Dietary fat reduction in postmenopausal women with primary breast cancer: Phase III Women's Intervention Nutrition Study (WINS)	2437	Relapse free survival	Cancer measurement	Secondary prevention/survivorship	Not found
Effects of Single Nucleotide Polymorphisms and Mediterranean Diet in Overweight or Obese Postmenopausal Women With Breast Cancer Receiving Adjuvant Hormone Therapy: A Pilot Randomized Controlled Trial	78	Weight loss, modification of nutrient intake, and metabolic parameters	Weight/Composition/Muscle	Secondary prevention/survivorship	35845810
Preoperative Immunonutrition in Liver Resection for Cancer: Results of the PROPILS Trial, a Multicenter Randomized Controlled Phase IV Trial	399	Overall complications classified in grade 2-3-4 or 5 by DINDO-CLAVIEN	Postoperative Misc endpoint	Adjuvant	Not found
Changes in dietary habits of women with breast cancer: preliminary results of a randomized controlled trial	35	Dietary intake and adherence	Complaince/Adherence/Feasibility	Adjuvant	Not found
A ketogenic diet reduces central obesity and serum insulin in women with ovarian or endometrial cancer	73	Fasting insulin, glucose, and beta-hydroxybuterate	Weight/Composition/Muscle	Misc/Mixed	30137481
Effect of nutritional counseling combined with oral nutritional supplements on clinical outcome of esophageal cancer patients under radiotherapy treatment	80	Energy intake and markers of nutritional status	Non-standard Quality of Life	Adjuvant	Not found
Impact of glutamine, eicosapntemacnioc acid, branched-chain amino acid supplements on nutritional status and treatment compliance of esophageal cancer patients on concurrent chemoradiotherapy and gastric cancer patients on chemotherapy	104	Body compositions, blood indicators of nutritional status, the incidence of complications, and completion rates of therapy	Weight/Composition/Muscle	Adjuvant	26080850
Effect of oral nutritional supplementation on nutritional status and quality of life in patients with gastric cancer after operation (23 cases RCT observations)	25	Body mass and body mass index	Weight/Composition/Muscle	Adjuvant	Not found
Enteral nutrition with supplemental arginine, RNA, and omega-3 fatty acids in patients after operation: Immunologic, metabolic, and clinical outcome	85	Post-operative infections	Postoperative Misc endpoint	Adjuvant	1377838
A Randomized Double-Blind Placebo-Controlled Trial of Fruit and Vegetable Concentrates on Intermediate Biomarkers in Head and Neck Cancer	134	P27 expression	Translational/Basic Science Endpoint	Secondary prevention/survivorship	28102098
Fish oil supplementation and inflammatory response during neoadjuvant chemoradiation for rectal cancer: Results from a prospective, randomized, controlled trial	114	CRP, albumin, and Glasgow prognostic score	Translational/Basic Science Endpoint	Adjuvant	Not found

Preoperative Fasting Abbreviation With Whey Protein Reduces the Occurrence of Postoperative Complications in Patients With Head and Neck Cancer: A Randomized Clinical Trial	49	Postoperative complications	Postoperative Misc endpoint	Adjuvant	33373478
Fasting mimicking diet as an adjunct to neoadjuvant chemotherapy for breast cancer in the multicentre randomized phase 2 DIRECT trial	131	Grade 3/4 toxicity	Non-standard Quality of Life	Adjuvant	32576828
A randomized clinical trial with oral immunonutrition (ω3-enhanced formula vs. arginine-enhanced formula) in ambulatory head and neck cancer patients	73	Nutritional variables	Weight/Composition/Muscle	Adjuvant	15802904
Fish oil supplementation during chemotherapy increases posterior time to tumor progression in colorectal cancer	30	Time to disease progression	Cancer measurement	Adjuvant	26700096
Nutritional intervention contributes to the improvement of symptoms related to quality of life in breast cancer patients undergoing neoadjuvant chemotherapy: A randomized clinical trial	34	Quality of life EORTC	Quality of Life	Adjuvant	Not found
Nutrition therapy in cachectic cancer patients. The Tight Caloric Control (TiCaCo) pilot trial	20	Recovery of body composition after nutrition therapy	Weight/Composition/Muscle	Cachexia/malnutrition	Not found
Nutrition therapy promotes overall survival in cachectic cancer patients through a biophysical pathway: the ticaconco trial	60	Overall survival	Overall Survival	Adjuvant	Not found
Benefits of immunonutrition in patients with head and neck cancer receiving chemoradiation: A phase II randomized, double-blind study	110	Prevention of severe oral mucositis	Non-standard Quality of Life	Adjuvant	35007812
Results of a diet/exercise feasibility trial to prevent adverse body composition change in breast cancer patients on adjuvant chemotherapy	90	Change in body composition	Weight/Composition/Muscle	Adjuvant	18501061
Flaxseed supplementation (not dietary fat restriction) reduces prostate cancer proliferation rates in men presurgery	161	Tumor proliferation rate	Translational/Basic Science Endpoint	Adjuvant	19064574
Presurgical weight loss affects tumour traits and circulating biomarkers in men with prostate cancer	40	Adherence/retention	Complaince/Adherence/Feasibility	Adjuvant	28881355
Randomized trial of weight loss in primary breast cancer: Impact on body composition, circulating biomarkers and tumor characteristics	33	Weight change	Weight/Composition/Muscle	Adjuvant	31442303
Observation on application effect of TCM diet intervention in improving malnutrition in patients with end-stage liver cancer	120	Nutritional status	Weight/Composition/Muscle	Palliative	Not found
Effects of a high dose, aglycone-rich soy extract on prostate-specific antigen and serum isoflavone concentrations in men with localized prostate cancer	66	Serum PSA	Cancer measurement	Secondary prevention/survivorship	21058191
A Very-Low-Fat Vegan Diet Increases Intake of Protective Dietary Factors and Decreases Intake of Pathogenic Dietary Factors	93	Intake of protective and pathogenic factors of chronic diseases	Complaince/Adherence/Feasibility	Misc/Mixed	18237581
Effects of nutritional support in patients with colorectal cancer	628	Nutritional status and survival	Weight/Composition/Muscle	Palliative	18982745
Effect of adequate nutrition therapy in rectal carcinoma post-surgery patients	24	Serum CRP and blood glucose levels	Translational/Basic Science Endpoint	Adjuvant	Not found
A diet and exercise intervention during Chemotherapy for breast cancer	40	Feasibility	Complaince/Adherence/Feasibility	Adjuvant	22238561
In a randomized trial in prostate cancer patients, dietary protein restriction modifies markers of leptin and insulin signaling in plasma extracellular vesicles	38	Molecular mediators in extracellular vesicles (insulin sensitivity)	Translational/Basic Science Endpoint	Adjuvant	Not found
Oral Nutritional Supplementation in Cancer Patients Who Were Receiving Chemo/Chemoradiation Therapy: A Multicenter, Randomized Phase II Study	89	Body weight and percent fat-free mass	Weight/Composition/Muscle	Adjuvant	32363940
Reduced infections with perioperative immunonutrition in head and neck cancer: Exploratory results of a multicenter, prospective, randomized, double-blind study	298	Incidence of postoperative infectious complications	Postoperative Misc endpoint	Adjuvant	24182765
Perioperative nutritional support in patients undergoing hepatectomy for hepatocellular carcinoma	124	Postoperative morbidity (anthropometric, biochemical, and immunologic)	Postoperative Misc endpoint	Adjuvant	7969324
Total parenteral alimentation with glutamine supplementation for the prevention of common complications in patients undergoing hematopoietic stem cell transplantation	48	Post-BMT complications (mucositis, diarrhea, and infection)	Non-standard Quality of Life	Adjuvant	Not found
Effect of conjugated linoleic acid supplementation on quality of life in rectal cancer patients undergoing preoperative chemoradiotherapy	33	Quality of Life (EORTC QLQ-C30)	Quality of Life	Adjuvant	28523042
Effects of ω-3 supplementation on the nutritional status, immune, and inflammatory profiles of gastric cancer patients: A randomized controlled trial	83	Weight modification	Weight/Composition/Muscle	Adjuvant	30710885
Effects of a nutrition intervention on acute and late bowel symptoms and health-related quality of life up to 24 months post radiotherapy in patients with prostate cancer: a multicentre randomised controlled trial	180	Bowel symptoms (EORTC QLQ-C30)	Quality of Life	Adjuvant	31758324
Dynamic metabolic response of prostate cancer patients treated with ADT and low carb diet	57	PSA doubling time	Cancer measurement	Palliative	Not found
A Randomized Controlled Trial of a 6-Month Low-Carbohydrate Intervention on Disease Progression in Men with Recurrent Prostate Cancer: Carbohydrate and Prostate Study 2 (CAPS2)	35	Weight loss	Weight/Composition/Muscle	Misc/Mixed	33712498
Effect of postoperative diet nursing with patient involved on nutritional status of the patients with rectal cancer	88	Nutritional status	Weight/Composition/Muscle	Adjuvant	Not found

Effect of Targeted Nutritional Intervention on Intestinal Flora, Defecation Function, and Postoperative Complications in Patients Who Underwent Radical Resection of Rectal Carcinoma	136	Intestinal flora	Translational/Basic Science Endpoint	Adjuvant	Not found
Randomized comparison of cooked and noncooked diets in patients undergoing remission induction therapy for acute myeloid leukemia	153	Major infection	Non-standard Quality of Life	Adjuvant	18955453
Effect of Standardized Nutritional Intervention in Patients with Nasopharyngeal Carcinoma Receiving Radiotherapy Complicated with Diabetes Mellitus	100	Glucose level	Weight/Composition/Muscle	Adjuvant	35756497
Immune Nutrition in Head and Neck Cancer. A Double Blind Randomised Controlled Trial of Perioperative Immune Enhancing Feeds in Patients with Advanced Head and Neck Cancer	67	Postoperative infections	Postoperative Misc endpoint	Adjuvant	Not found
A randomized controlled trial of preoperative oral supplementation with a specialized diet in patients with gastrointestinal cancer	305	Reduction of postoperative infection rate and length of postoperative stay	Postoperative Misc endpoint	Adjuvant	12055582
Route and composition of postoperative nutritional support: Impact on immune-metabolic response and postoperative outcome	260	Immune response	Translational/Basic Science Endpoint	Adjuvant	Not found
Short-term preoperative supplementation of an immunoenriched diet does not improve clinical outcome in well-nourished patients undergoing abdominal cancer surgery	108	Overall rate of postoperative complications	Postoperative Misc endpoint	Adjuvant	Not found
Evaluation of perioperative nutritional therapy in patients with gastrointestinal tract neoplasms	50	Gastrointestinal and infectious complications and length of hospital stay	Postoperative Misc endpoint	Adjuvant	22072355
Efficacy evaluation of an oral powder supplement enriched with eicosapentaenoic acid in cancer patients	61	Anthropometric measurements and nutritional status	Weight/Composition/Muscle	Misc/Mixed	22411387
A combination of tomato and soy products for men with recurring prostate cancer and rising prostate specific antigen	41	Compliance	Complaince/Adherence/Feasibility	Misc/Mixed	18444145
Effects of a dietary intervention to increase omega-3 intake compared to dutasteride in men with low-risk prostate cancer under active surveillance: Preliminary results on fatty acid intake and fatty acid profiles of red blood cells and prostate tissue	120	Omega-3 dietary intake, fatty acid profiles of red blood cells, and prostate tissue	Translational/Basic Science Endpoint	Secondary prevention/survivorship	Not found
The effect of immunonutrition on tumor infiltrative t lymphocytes and regulatory t cells in rectal tumor patients receiving neoadjuvant chemoradiotherapy: a prospective randomized clinical study	40	Tumor-infiltrating lymphocytes, and regulatory T cells	Translational/Basic Science Endpoint	Adjuvant	36326418
Effects of emotional intervention and Chinese medicated diet in improving the quality of life in patients with liver cancer after chemotherapy	94	Quality of life (unspecified scale)	Non-standard Quality of Life	Adjuvant	Not found
A Pilot Randomised Controlled Trial Examining the Benefit of a Neutropenic Diet for Children Undergoing Cancer Treatment	42	Febrile neutropenia rate	Non-standard Quality of Life	Adjuvant	35225115
Assessment of Nutritional Status in Children with Cancer and Effectiveness of Oral Nutritional Supplements	45	Anthropometric mesurements and biochemical parameters (serum albumin, prealbumin, and total protein)	Weight/Composition/Muscle	Misc/Mixed	26418028
Application of Glutamine-enriched nutrition therapy in childhood acute lymphoblastic leukemia	48	Indicators of general nutritional status such as weight, height, and triceps skinfold thickness	Weight/Composition/Muscle	Adjuvant	27401338
Prospective randomized investigation implementing immunonutritional therapy using a nutritional supplement with a high blend ratio of ω-3 fatty acids during the perioperative period for head and neck carcinomas	28	Nutritional status (anthropometric measurement)	Weight/Composition/Muscle	Adjuvant	29420749
Miami NICE trial: Nutritional support for patients incurring chemotherapy side effects	50	Change in BMI	Weight/Composition/Muscle	Misc/Mixed	Not found
Randomized controlled clinical trial assessing the effects of oral nutritional supplements in postoperative gastric cancer patients	157	Postoperative body weight loss	Postoperative Misc endpoint	Adjuvant	27807617
Change in women's diet and body mass following intensive intervention for early-stage breast cancer	180	Dietary intake and body mass	Weight/Composition/Muscle	Adjuvant	11320947
Immunonutrition in elective gastrointestinal surgery patients	100	Postoperative complications	Postoperative Misc endpoint	Adjuvant	17461312
Dietary intervention as adjuvant therapy in breast cancer patients - a feasibility study	240	Daily energy and nutrient intake	Complaince/Adherence/Feasibility	Adjuvant	2265258
A double-blind randomized controlled trial of the effects of eicosapentaenoic acid supplementation on muscle inflammation and physical function in patients undergoing colorectal cancer resection	64	Cardiorespiratory fitness, physical strength, and lean muscle mass	Weight/Composition/Muscle	Adjuvant	31648815
Efficacy of Omega Fatty Acid Supplementation on mRNA Expression Level of Tumor Necrosis Factor Alpha in Patients with Gastric Adenocarcinoma	34	TNF-alpha mRNA expression	Translational/Basic Science Endpoint	Adjuvant	27170003
A prospective randomized controlled trial on the value of prophylactic oral nutritional supplementation in locally advanced nasopharyngeal carcinoma patients receiving chemo-radiotherapy	114	Nutritional status and weight loss	Weight/Composition/Muscle	Adjuvant	33032180
The impact of nutrition support on body composition in cancer outpatients receiving radiotherapy	36	Body weight and composition	Weight/Composition/Muscle	Adjuvant	15226773
Oral nutritional support can shorten the duration of parenteral hydration in end-of-life cancer patients: A randomized controlled trial	27	Drip infusion in vein (DIV)-free survival	Non-standard Quality of Life	Palliative	25437180
Efficacy of EPA-enriched supplement compared with standard formula on body weight changes in malnourished patients with head and neck cancer undergone surgery: a randomized study	65	Perioperative weight changes	Weight/Composition/Muscle	Cachexia/malnutrition	31647147

An eicosapentaenoic acid supplement versus megestrol acetate versus both for patients with cancer-associated wasting: A North Central Cancer Treatment Group and National Cancer Institute of Canada collaborative effort	421	10% weight gain above baseline	Weight/Composition/Muscle	Cachexia/malnutrition	15197210
Structured triglycerides were well tolerated and induced after hepatectomy in patients with hepatocarcinoma	49	Liver functions, biochemical variables, and postoperative complications	Translational/Basic Science Endpoint	Adjuvant	Not found
Effect of glutamine's nutrition support on the postoperative nutrition and immune function in malignant tumor of gynecology patients	60	Postoperative nutrition and immune function	Postoperative Misc endpoint	Adjuvant	25016261
Benefits of Oral Nutritional Supplements in Patients with Locally Advanced Nasopharyngeal Cancer during Concurrent Chemoradiotherapy: An Exploratory Prospective Randomized Trial	100	Body weight, BMI, and nutritional status	Weight/Composition/Muscle	Adjuvant	30633580
Vitamin d supplementation and disease-free survival in stage ii melanoma: A randomized placebo controlled trial	104	Disease free survival	Cancer measurement	Secondary prevention/survivorship	34199802
Muscle mass, strength, and index in various models of preoperative immunonutrition in invasive gastric cancer patients	46	Lean body mass, muscle strength, and skeletal muscle mass and index	Weight/Composition/Muscle	Adjuvant	Not found
Preoperative nutritional support in cancer patients with no clinical signs of malnutrition—prospective randomized controlled trial	113	Nutritional status measured by change in weight and lab parameters	Weight/Composition/Muscle	Adjuvant	25091056
Phagocytic activity of blood platelets in various models of preoperative oral immunonutrition in invasive cancer patients	52	Phagocytic activity of thrombocytes	Translational/Basic Science Endpoint	Adjuvant	Not found
Lymphocyte subpopulation in a model of preoperative oral and parenteral glutamine-based immunonutrition in patients with invasive gastric cancer	46	Total content of lymphocytes and their subpopulations	Translational/Basic Science Endpoint	Adjuvant	Not found
Efficacy of perioperative immunonutrition in esophageal cancer patients undergoing esophagectomy	40	Number of infectious complications	Postoperative Misc endpoint	Adjuvant	30468936
A Prospective Randomized Controlled Trial to Study the Impact of a Nutrition-Sensitive Intervention on Adult Women with Cancer Cachexia Undergoing Palliative Care in India	63	Body weight	Weight/Composition/Muscle	Cachexia/malnutrition	Not found
Randomized study of prevention of gastrointestinal toxicities by nutritional support using an amino acid-rich elemental diet during chemotherapy in patients with esophageal cancer (KDOG 1101)	71	Incidence of grade 2 or higher GI toxicity	Non-standard Quality of Life	Adjuvant	33009977
Effects of 4 weeks of Lactobacillus plantarum 299v supplementation on nutritional status, enteral nutrition tolerance, and quality of life in cancer patients receiving home enteral nutrition - A double-blind, randomized, and placebo-controlled trial	35	Nutritional status	Weight/Composition/Muscle	Misc/Mixed	33015813
Early postoperative enteral nutrition with arginine-ω-3 fatty acids and ribonucleic acid-supplemented diet versus placebo in cancer patients: An immunologic evaluation of Impact®	42	Postoperative immunologic responses	Translational/Basic Science Endpoint	Adjuvant	7536138
Early enteral feeding in postsurgical cancer patients. Fish oil structured lipid-based polymeric formula versus a standard polymeric formula	50	Safety and tolerance	Complaince/Adherence/Feasibility	Adjuvant	8604913
Effects of Ketogenic metabolic therapy on patients with breast cancer: A randomized controlled clinical trial	80	Response rate	Cancer measurement	Adjuvant	Not found
A Randomized Clinical Trial of Preoperative Administration of Branched-Chain Amino Acids to Prevent Postoperative Ascites in Patients with Liver Resection for Hepatocellular Carcinoma	77	Development for postoperative refractory ascites	Postoperative Misc endpoint	Adjuvant	27338747
The impact of nutritional support on treatmentrelated complications, qol and survival in lung cancer patients undergoing radiotherapy: A randomized, controlled study	45	Toxicity	Non-standard Quality of Life	Adjuvant	Not found
The effects of patient participation-based dietary intervention on nutritional and functional status for patients with gastrectomy: A randomized controlled trial	56	Cognitive, affective, behavioral, physical, and functional outcomes	Non-standard Quality of Life	Adjuvant	23632471
The Effect of Nutrition Intervention with Oral Nutritional Supplements on Pancreatic and Bile Duct Cancer Patients Undergoing Chemotherapy	34	Anthropometric measures (weight, composition, and PG SGA)	Weight/Composition/Muscle	Palliative	31121926
The effect of preoperative nutritional supports on patients with gastrointestinal cancer: Prospective randomized study	100	Total antioxidant capacity	Translational/Basic Science Endpoint	Adjuvant	22260826
The Effects of a Mediterranean Diet Intervention on Cancer-Related Fatigue for Patients Undergoing Chemotherapy: A Pilot Randomized Controlled Trial	33	Feasibility	Complaince/Adherence/Feasibility	Adjuvant	36077737
The impact of immunostimulating nutrition on infectious complications after upper gastrointestinal surgery: A prospective, randomized, clinical trial	205	Both surgical and non-surgical complications	Postoperative Misc endpoint	Adjuvant	18650630
Standard and immunomodulating enteral nutrition in patients after extended gastrointestinal surgery--a prospective, randomized, controlled clinical trial	196	Number and type of complications and length of hospital stay	Postoperative Misc endpoint	Adjuvant	18571296
L-Carnitine-supplementation in advanced pancreatic cancer (CARPAN) - A randomized multicentre trial	72	TNF alpha and l-Carnitine level	Translational/Basic Science Endpoint	Palliative	22824168
Dietary intervention in prostate cancer patients: PSA response in a randomized double-blind placebo-controlled study	37	Rise in PSA	Cancer measurement	Misc/Mixed	Not found

Long term improved quality of life by a 2-week group physical and educational intervention shortly after breast cancer chemotherapy completion. Results of the 'Programme of Accompanying women after breast Cancer treatment completion in Thermal resorts' (PACThe) randomised clinical trial of 251 patients	251	Quality of life-SF 36	Quality of Life	Adjuvant	23352440
Influence of pre-operative oral carbohydrate loading vs. standard fasting on tumor proliferation and clinical outcome in breast cancer patients ─ a randomized trial	61	Mitotic activity index	Translational/Basic Science Endpoint	Adjuvant	31703648
Nursing Effect and Prognosis of Perioperative Nutritional Support Therapy for Severe Malnutrition Patients with Colorectal Cancer	127	Post-operative recovery (exhaustion time and hospitalization time)	Postoperative Misc endpoint	Cachexia/malnutrition	Not found
Early enteral immunonutrition support protects the cellular and humoral immune functions of patients with pancreatic cancer after chemotherapy	78	Whole blood CD4+ and CD8+, IgG, IgA, and IgM	Translational/Basic Science Endpoint	Misc/Mixed	Not found
The effect of low-nitrogen and low-calorie parenteral nutrition combined with enteral nutrition on inflammatory cytokines and immune functions in patients with gastric cancer: a double blind placebo trial	90	Levels of IgG, IgA and IgM, IL-2, CRP, and TNFa	Translational/Basic Science Endpoint	Adjuvant	25967707
A randomized nutrition counseling intervention in pediatric leukemia patients receiving steroids results in reduced caloric intake	22	Caloric intake	Complaince/Adherence/Feasibility	Adjuvant	27615542
Enteral immunonutrition versus enteral nutrition for patients undergoing esophagectomy: a double-blinded randomized controlled trial	99	Immune indicators	Translational/Basic Science Endpoint	Adjuvant	Not found
Feasibility of a low-fat/high-fiber diet intervention with soy supplementation in prostate cancer patients after prostatectomy	40	Compliance at 4 years	Complaince/Adherence/Feasibility	Secondary prevention/survivorship	17392697
A multidisciplinary team approach for nutritional interventions conducted by specialist nurses in patients with advanced colorectal cancer undergoing chemotherapy: A clinical trial	110	Patient weight, serum albumin, and prealbumin levels	Weight/Composition/Muscle	Misc/Mixed	28658162
Effects of Zinc Supplementation on Clinical Outcomes in Patients Receiving Radiotherapy for Head and Neck Cancers: A Double-Blinded Randomized Study	97	Overall survival, disease-free, metastases-free survival rates, and local free survival	Overall Survival	Adjuvant	17980503
Effect of Psychological Intervention Combined with Dietary Guidance on Quality of Life and Long-Term Efficacy of Bushen Quyu Decoction in Treatment of Patients with Advanced Ovarian Cancer	220	Quality of life and clinical efficacy according to evaluation criteria for treatment efficacy of solid tumors	Non-standard Quality of Life	Misc/Mixed	34733335
An enhanced nutritional support pathway including extended preoperative and home enteral nutrition is safe, feasible, and may benefit patients undergiong enhanced recovery after esophagectomy: A pilot randomized clinical trial	63	Feasibility	Complaince/Adherence/Feasibility	Adjuvant	Not found
A Randomized Study on the Effect of Metformin Combined with Intensive-Exercise Diet Therapy on Glucose and Lipid Metabolism and Islet Function in Patients with Renal Cell Carcinoma and Diabetes	120	Fasting blood glucose, HbA1c, total cholesterol, HDL, LDL, PFS	Translational/Basic Science Endpoint	Secondary prevention/survivorship	Not found
Early postoperative jejunostomy feeding with an immune modulating diet in patients undergoing resectional surgery for upper gastrointestinal cancer: a prospective, randomized, controlled, double-blind study	120	Development of infective complications	Postoperative Misc endpoint	Adjuvant	Not found
Feasibility and efficacy of progressive resistance training and dietary supplements in radiotherapy treated head and neck cancer patients-the DAHANCA 25A study	30	Lean body mass percent	Weight/Composition/Muscle	Adjuvant	23190359
Analysis of the Effect of Exercise Combined with Diet Intervention on Postoperative Quality of Life of Breast Cancer Patients	104	Nutritional endpoint for quality of life (SF-6 & QLQ-C30)	Non-standard Quality of Life	Adjuvant	35669367
Palliative Nutritional Intervention in Addition to Cyclooxygenase and Erythropoietin Treatment for Patients with Malignant Disease: Effects on Survival, Metabolism, and Function: A Randomized Prospective Study	220	Food intake, energy balance, body composition, and maximum exercise capacity	Weight/Composition/Muscle	Adjuvant	15112279
The effect of zinc sulfate in the prevention of high-dose chemotherapy-induced mucositis: a double-blind, randomized, placebo-controlled study	60	Severity of mucositis	Non-standard Quality of Life	Adjuvant	21692101
Immunonutrition to improve the quality of life of upper gastrointestinal cancer patients undergoing neoadjuvant treatment prior to surgery (NEOIMMUNE): Double blind randomized controlled multi-center clinical trial	300	QOL as measured by the EORTC-QLQ-C30	Quality of Life	Adjuvant	Not found
Ketogenic diets as an adjuvant therapy for glioblastoma (KEATING): a randomized, mixed methods, feasibility study	12	Compliance and retention	Complaince/Adherence/Feasibility	Adjuvant	Not found
An exercise and nutrition intervention for ovarian cancer patients during and after first-line chemotherapy (BENITA study): A randomized controlled pilot trial	15	Recruitment rate, adherence to intervention, and completion rate	Complaince/Adherence/Feasibility	Adjuvant	35197344
The effect of nutritional supplements on food intake in patients undergoing radiotherapy	40	Protein and caloric intake	Complaince/Adherence/Feasibility	Adjuvant	10382188
Is supplementation with elemental diet feasible in patients undergoing pelvic radiotherapy?	50	Compliance	Complaince/Adherence/Feasibility	Adjuvant	16289498
Effect of preoperative immunonutrition in patients undergoing hepatectomy; a randomized controlled trial	26	WBC, EPA, Alb, Pre alb, AST, ALT, TG, and FFA	Translational/Basic Science Endpoint	Adjuvant	30741015

Efficacy of prolonged elemental diet therapy after pancreaticoduodenectomy for pancreatic ductal adenocarcinoma: A pilot prospective randomized trial (UMIN000004108)	39	Complications necessitating readmission after discharge	Postoperative Misc endpoint	Adjuvant	31677701
Effects of medical nutrition therapy compared with general nutritional advice on nutritional status and nutrition-related complications in esophageal cancer patients receiving concurrent chemoradiation: A randomized controlled trial	100	Nutritonal status measured by PG-SGA	Non-standard Quality of Life	Adjuvant	Not found
Perioperative standard oral nutrition supplements versus immunonutrition in patients undergoing colorectal resection in an Enhanced Recovery (ERAS) protocol	244	Overall postoperative 30-day morbidity rate	Postoperative Misc endpoint	Adjuvant	Not found
Multicenter, phase III trial comparing selenium supplementation with observation in gynecologic radiation oncology: Follow-up analysis of the survival data 6 years after cessation of randomization	81	Efficiency of selenium supplementation	Complaince/Adherence/Feasibility	Adjuvant	25015649
Effects of dietary supplements of pediasure and carnitine on the anthropometric indices in children with acute lymphoblastic leukemia under chemotherapy, in Afzalipour Hospital, Kerman, Iran	34	Anthropometric indices	Weight/Composition/Muscle	Adjuvant	Not found
Impact of nutritional supplementation on treatment delay and morbidity in patients with head and neck tumors treated with irradiation	23	Body weight and tricep skin-fold thickness	Weight/Composition/Muscle	Adjuvant	1562782
Parenteral nutrition effects of Omega-3 fatty acids on C-reactive protein, high-density lipoprotein, lymphocyte characteristics and the treatment of critically ill patients	80	C-reactive protein, HDL, and lymphocyte characteristics	Translational/Basic Science Endpoint	Misc/Mixed	32538747
Effects of branched-chain amino acid supplementation after radiofrequency ablation for hepatocellular carcinoma: A randomized trial	51	Time until distant recurrence of HCC	Cancer measurement	Adjuvant	27908546
Prostatic concentration of soy isoflavone exceeds serum after dietary supplementation	25	Tissue and serum isoflavone concentration	Translational/Basic Science Endpoint	Adjuvant	
L-Alanin-L-Glutamine supplementation improves the outcome after colorectal surgery for cancer	109	Postoperative complications	Postoperative Misc endpoint	Adjuvant	17573745
Oral supplementation with carbohydrate- and branched-chain amino acid-enriched nutrients improves postoperative quality of life in patients undergoing hepatic resection	76	Postoperative QoL using short-form 36 (SF-36) health questionnaire	Quality of Life	Adjuvant	20852905
Attenuation of the systemic inflammatory response and infectious complications after gastrectomy with preoperative oral arginine and ω-3 fatty acids supplemented immunonutrition	60	Postoperative outcome	Postoperative Misc endpoint	Adjuvant	19629583
Nutritional behaviour and quality of life during oncological polychemotherapy: Results of a prospective study on the efficacy of oral nutrition therapy in patients with acute leukaemia	29	Percentage of patients with initial nutritional status before antineoplastic therapy	Weight/Composition/Muscle	Adjuvant	1425861
Nutritional supplementation with an immuneenhancing formula in the patients with esophageal cancer	97	CD3 lymph(%), CD4 lymph(%), CD8 lymph(%), and CD4/CD8 ratio	Translational/Basic Science Endpoint	Adjuvant	Not found
Dramatic dietary fat reduction is feasible for breast cancer patients: Results of the randomised study, WINS (UK) - stage 1	190	Fat intake reduction	Complaince/Adherence/Feasibility	Secondary prevention/survivorship	21868187
The Men's Eating and Living (MEAL) Study: A Cancer and Leukemia Group B Pilot Trial of Dietary Intervention for the Treatment of Prostate Cancer	74	Differences in dietary intakes and plasma carotenoid concentrations	Translational/Basic Science Endpoint	Misc/Mixed	Not found
A randomized pilot trial of dietary modification for the chemoprevention of noninvasive bladder cancer: The Dietary Intervention in Bladder Cancer Study	48	Changes in dietary biomarkers (alpha-carotene and urinary isothyocyanates)	Translational/Basic Science Endpoint	Secondary prevention/survivorship	23867158
Effect of a Behavioral Intervention to Increase Vegetable Consumption on Cancer Progression Among Men With Early-Stage Prostate Cancer: The MEAL Randomized Clinical Trial	478	Time to progression measured by PSA	Cancer measurement	Secondary prevention/survivorship	32586177
A randomized study of nutritional support in patients with colorectal and gastric cancer	137	QoL (EORTC-QLQ C-30)	Quality of Life	Misc/Mixed	12235650
Effects of a dietary intervention on acute gastrointestinal side effects and other aspects of health-related quality of life: A randomized controlled trial in prostate cancer patients undergoing radiotherapy	130	EORTC QLQ-C30 and QLQ-PR25	Quality of Life	Adjuvant	22633817
Shrinking preoperative fast time with maltodextrin and protein hydrolysate in gastrointestinal resections due to cancer	22	Postoperative hospital length of stay	Postoperative Misc endpoint	Adjuvant	23759267
Influence of a diet very high in vegetables, fruit, and fiber and low in fat on prognosis following treatment for breast cancer: the Women's Healthy Eating and Living (WHEL) randomized trial	3088	Invasive breast cancer recurrence or new primary breast cancer and death due to any cause	Cancer measurement	Secondary prevention/survivorship	17635889
Long-term oral branched chain amino acids in patients undergoing chemoembolization for hepatocellular carcinoma: A randomized trial	84	Post-TACE morbidity	Non-standard Quality of Life	Adjuvant	15043519
Nutritional counseling in patients with head, neck and esophagus cancer in (chemo)radiotherapy	29	Weight loss	Weight/Composition/Muscle	Adjuvant	Not found
Randomized trial of the effects of individual nutritional counseling in cancer patients	61	Reduced weight loss	Weight/Composition/Muscle	Adjuvant	24269077
Efficacy of ready-to-use therapeutic food in malnourished children with cancer: Results of a randomized, open-label phase 3 trial	260	Weight gain >10% at 6 weeks	Weight/Composition/Muscle	Cachexia/malnutrition	34212474
Postoperative early enteral nutrition therapy with long peptide mixture for gastric cancer patients: A clinical control study	70	Recovery time of intestinal peristalsis and ventilation time	Postoperative Misc endpoint	Adjuvant	Not found
Effect of whole-course nutrition management on patients with esophageal cancer undergoing concurrent chemoradiotherapy: A randomized control trial	96	Nutritional status via PG-SGA & BMI	Weight/Composition/Muscle	Adjuvant	31526964
Neutropenic versus regular diet for acute leukaemia induction chemotherapy: randomised controlled trial	200	Infection rate	Non-standard Quality of Life	Adjuvant	35803707

Association between amino acids, biomarkers of prostate cancer and inflammation in norwegian prostate cancer patients	74	PSA	Cancer measurement	Adjuvant	Not found
Individualized nutrition intervention is of major benefit to colorectal cancer patients: Long-term follow-up of a randomized controlled trial of nutritional therapy	111	Body weight	Weight/Composition/Muscle	Adjuvant	23134880
Effect of a specific supplement enriched with n-3 polyunsaturated fatty acids on markers of inflammation, oxidativestress and metabolic status of ear, nose and throat cancer patients	26	Inflammation, oxidative stress, and metabolic status (total antioxidant capacity)	Translational/Basic Science Endpoint	Adjuvant	Not found
Effect of Early Peripheral Parenteral Nutrition Support in an Enhanced Recovery Program for Colorectal Cancer Surgery: A Randomized Open Trial	158	Incidence of postoperative complications (morbi-mortality for 30 days)	Postoperative Misc endpoint	Adjuvant	Not found
Effects of an oral nutritional supplement containing eicosapentaenoic acid on nutritional and clinical outcomes in patients with advanced non-small cell lung cancer: randomised trial	112	Weight, body composition, dietary intake, inflammatory parameters, HRQL	Weight/Composition/Muscle	Palliative	24746976
Effects of a Plant-Based High-Protein Diet on Fatigue in Breast Cancer Patients Undergoing Adjuvant Chemotherapy–a Randomized Controlled Trial	103	Self reported CRF which was assessed using the Fatigue Symptom Inventory	Non-standard Quality of Life	Adjuvant	36546552
Randomized, double-blind, controlled study of glycyl-glutamine-dipeptide in the parenteral nutrition of patients with acute leukemia undergoing intensive chemotherapy	30	Duration of neutropenic fever as a proportion of neutropenia	Non-standard Quality of Life	Adjuvant	14990264
Randomized, double-blind, placebo-controlled crossover study in men with prostate cancer and rising PSA: Effectiveness of a dietary supplement	49	Rate of increase of PSA	Cancer measurement	Misc/Mixed	16263208
Effects of an exercise and hypocaloric healthy eating program on biomarkers associated with long-term prognosis after early-stage breast cancer: A randomized controlled trial	90	Change in body weight and composition	Weight/Composition/Muscle	Secondary prevention/survivorship	23184120
Effect of a Perioperative Nutritional Supplementation with Oral Impact® in Patients undergoing Hepatic Surgery for Liver Cancer: A Prospective, Placebo-Controlled, Randomized, Double-Blind Study	35	Factor V, 3 days after surgery	Translational/Basic Science Endpoint	Adjuvant	27007018
Preoperative oral supplementation with long-chain Ω-3 fatty acids beneficially alters phospholipid fatty acid patterns in liver, gut mucosa, and tumor tissue	40	EPA and DHA in liver tissue, gut mucosa, and tumor tissue	Translational/Basic Science Endpoint	Adjuvant	15961678
Comprehensive Diet and Exercise Intervention During Radiation Therapy Can Improve Metabolic and Inflammatory Markers in Breast Cancer Patients	43	Positive influence on body composition, HbA1C, adiponecting, leptin, and insulin	Translational/Basic Science Endpoint	Adjuvant	Not found
To compare outcome of Intensive nutritional support with standard practise in head ands neck cancer	60	Protein and kilocalorie intake	Weight/Composition/Muscle	Adjuvant	Not found
Chinese food therapy combined with chemotherapy in the treatment of advanced ovarian cancer based on	74	Treatment quality of life	Non-standard Quality of Life	Adjuvant	Not found
Randomized controlled trial comparing a low-fat diet with a weight-reduction diet in breast cancer-related lymphedema	51	Arm volume at 24 weeks	Weight/Composition/Muscle	Misc/Mixed	17393377
Application of the Diet and Nutrition Management System in the Nutrition Management of Tumor Patients Treated with Chemotherapy	86	Awareness rate of disease knowledge	Non-standard Quality of Life	Misc/Mixed	Not found
Influence of nutritional support on tolerability and results of treatment in patients with newly diagnosed hemoblastoses received program chemotherapy	40	Serum albumin and frequency of constipation	Translational/Basic Science Endpoint	Adjuvant	Not found
Effect of early nutritional support on the clinical efficacy of gastrointestinal cancer patients	80	Nutritional status	Weight/Composition/Muscle	Adjuvant	Not found
Phase II randomized controlled trial (RCT) of medical intensive nutrition therapy (MINT) to improve chemotherapy (CT) tolerability in malnourished patients with solid tumor malignancies	186	Chemotherapeutic associated toxicity	Non-standard Quality of Life	Cachexia/malnutrition	Not found
Potential benefits of early nutritional intervention in adults with upper gastrointestinal cancer: a pilot randomised trial	21	Quality of Life (EORTC QLQ-C30)	Quality of Life	Adjuvant	Not found
Anorexia-cachexia syndrome in cancer patients: Pathogenetic aspects and treatment options	96	Reduction in weight loss	Weight/Composition/Muscle	Adjuvant	Not found
Reduced postoperative infections with an immune-enhancing nutritional supplement	124	LOS (excluding rehabilitation stay)	Postoperative Misc endpoint	Adjuvant	10369282
Randomised controlled trial of one week strict low-iodine diet versus one week non-specified low iodine diet in differentiated thyroid carcinoma	104	Urinary iodine level	Translational/Basic Science Endpoint	Adjuvant	Not found
Rapid Incorporation of ω-3 Fatty Acids into Colonic Tissue after Oral Supplementation in Patients with Colorectal Cancer: A Randomized, Placebo-Controlled Intervention Trial	148	Colonic wall content of EPA, docosapentaenoic acid (DPAn-3), and DHA	Translational/Basic Science Endpoint	Adjuvant	23788002
Effect of a leucine-rich supplement in combination with nutrition and physical exercise in advanced cancer patients: A randomized controlled intervention trial	52	Short physical performance battery	Non-standard Quality of Life	Palliative	32340904
Effect of targeted nutrition intervention on defecation and postoperative complications in patients undergoing radical resection for rectal cancer	130	Defecation function	Non-standard Quality of Life	Adjuvant	Not found
A randomized, double blind comparative study of prophylactic parenteral nutritional support with or without glutamine in autologous stem cell transplantation for hematological malignancies - Three years' follow-up	44	Overall survival	Overall Survival	Adjuvant	16284692
Randomized Controlled Trial Investigating Perioperative Immunonutrition for Patients Undergoing Cytoreductive Surgery (CRS) and Hyperthermic Intraperitoneal Chemotherapy (HIPEC)	62	Length of hospitalization stay	Postoperative Misc endpoint	Adjuvant	36394687

Effect of different nutritional support modes on humoral immunity and outcomes after esophagectomy	46	Infection-related complications and hospital stay	Postoperative Misc endpoint	Adjuvant	Not found
Evolution of inflammatory markers in patients with advanced head and neck carcinoma with prophylactic percutaneous gastrostomy	38	Inflammatory profile (markers)	Translational/Basic Science Endpoint	Adjuvant	Not found
Preoperative nutritional support for patients undergoing elective colorectal cancer surgery - does it really work?	120	Changes in muscle strength, muscle mass, and self-sufficiency	Weight/Composition/Muscle	Adjuvant	35258042
A double blind, placebo controlled randomised trial (RCT) evaluating the effect of a polyphenol rich whole food supplement on PSA progression in men	203	Median rise in PSA	Cancer measurement	Adjuvant	Not found
Longitudinal changes in body weight and body composition among women previously treated for breast cancer consuming a high-vegetable, fruit and fiber, low-fat diet	77	Longitudinal changes in intake, body weight, waist:hip ratio, BMI, and body composition	Weight/Composition/Muscle	Secondary prevention/survivorship	15309460
beta-carotene supplementation in patients radically treated for stage I-II head and neck cancer: results of a randomized trial	214	Survival and disease-free survival	Overall Survival	Adjuvant	14534715
Transcriptional changes in prostate of men on active surveillance after a 12-mo glucoraphanin-rich broccoli intervention-results from the Effect of Sulforaphane on Prostate CAncer PrEvention (ESCAPE) randomized controlled trial	61	Changes in gene expression	Translational/Basic Science Endpoint	Secondary prevention/survivorship	30982861
Oral administration of the amino acids cystine and theanine attenuates the adverse events of S-1 adjuvant chemotherapy in gastrointestinal cancer patients	70	Adverse effects with S-1 adjuvant chemotherapy	Non-standard Quality of Life	Adjuvant	27306219
Perioperative immunonutrition in well-nourished patients undergoing surgery for head and neck cancer: Evaluation of inflammatory and immunologic outcomes	8	Postoperative immune and inflammatory status (C-reactive protein, total lymphocytes, CD3, CD4, CD8, immunoglobulin G, etc.)	Translational/Basic Science Endpoint	Adjuvant	23571650
Glutamine-enriched nutrition does not reduce mucosal morbidity or complications after stem-cell transplantation for childhood malignancies: A prospective randomized study	120	Mucositis	Non-standard Quality of Life	Adjuvant	21499196
Influence of a nutritional intervention on dietary intake and quality of life in cancer patients: A randomized controlled trial	58	QoL and dietary intake	Quality of Life	Misc/Mixed	24103511
Effects of nutrition and physical exercise intervention in palliative cancer patients: A randomized controlled trial	58	QoL (EORTC-QLQ C-30)	Quality of Life	Palliative	28651827
Calorie restriction and synbiotics effect on quality of life and edema reduction in breast cancer-related lymphedema, a clinical trial	135	Differences in quality of life score (Lymphedema Life Impact Scale)	Non-standard Quality of Life	Misc/Mixed	32898787
Randomized phase 2 study of gemcitabine and cisplatin with or without vitamin supplementation in patients with advanced esophagogastric cancer	82	Response rate	Cancer measurement	Palliative	29696360
Immunonutrition improves functional capacities in head and neck and esophageal cancer patients undergoing radiochemotherapy: A randomized clinical trial	37	Functional outcomes	Non-standard Quality of Life	Adjuvant	23849811
Preventing weight gain during adjuvant chemotherapy for breast cancer: A dietary intervention study	96	Anthropometric changes	Weight/Composition/Muscle	Adjuvant	22869285
ERGO2: A prospective randomized trial of a 9-day schedule of calorically restricted ketogenic diet and fasting or standard diet in addition to re-irradiation for malignant glioma	50	Progression free survival rate	Cancer measurement	Adjuvant	32619561
Effect of glutamine-enriched nutritional support on intestinal mucosal barrier function, MMP-2, MMP-9 and immune function in patients with advanced gastric cancer during perioperative chemotherapy	94	Lactulose/mannitol ratio, D-lactate levels, MMP-9, MMP-2, CD3+, CD4+, CD8+, CD4+/CD8+, and immunoglobulins	Translational/Basic Science Endpoint	Adjuvant	28927119
Effects of glutamine on plasma endotoxin and immune function in postperative patients with laryngeal carcinoma	39	Plasma endotoxin level and immune function	Translational/Basic Science Endpoint	Adjuvant	Not found
Clinical trial: Efficacy of a low or modified fat diet for the prevention of gastrointestinal toxicity in patients receiving radiotherapy treatment for pelvic malignancies	117	Difference in change in Inflammatory Bowel Disease Questionnaire – Bowel (IBDQ-B) score	Non-standard Quality of Life	Adjuvant	22515941
High fibre diet, low fibre diet or habitual diet for the prevention of radiation-induced toxicity in pelvic cancer: A multi-centre randomised controlled trial	166	Change in Inflammatory Bowel Disease Questionnaire – Bowel Subset (IBDQ-B) score	Non-standard Quality of Life	Adjuvant	Not found
Dietary supplementation with n-3-fatty acids in patients with pancreatic cancer and cachexia: marine phospholipids versus fish oil - a randomized controlled double-blind trial	60	Change of weight and appetite	Weight/Composition/Muscle	Misc/Mixed	28578704
Effects of Perioperative Oral Nutrition Supplementation in Malaysian Patients Undergoing Elective Surgery for Breast and Colorectal Cancers—A Randomised Controlled Trial	91	Body weight, BMI, and serum albumin level	Weight/Composition/Muscle	Adjuvant	35276977
A study of caloric restriction versus standard diet in overweight men with newly diagnosed prostate cancer: A randomized controlled trial	19	Changes in weight	Weight/Composition/Muscle	Adjuvant	23775525
A randomized controlled trial of postoperative artificial nutrition in malnourished patients with gastrointestinal cancer	646	Postoperative complications, mortality, and postoperative length of hospital stay	Postoperative Misc endpoint	Adjuvant	18000777

Preoperative enteral immunonutrition improves postoperative outcome in patients with gastrointestinal cancer	60	Postoperative serum antibody and complement levels	Translational/Basic Science Endpoint	Adjuvant	16830214
Effect of a nutritional therapy using a late evening snack for hepatocellular carcinoma	23	Blood biochemical data, nutrition status using an indirect calorimeter, and area under the concentration curve for glucose	Translational/Basic Science Endpoint	Adjuvant	Not found
Impact of Enhanced Recovery after Surgery with Preoperative Whey Protein-Infused Carbohydrate Loading and Postoperative Early Oral Feeding among Surgical Gynecologic Cancer Patients: An Open-Labelled Randomized Controlled Trial	118	Length of postoperative hospital stay, clear fluid toleration, food toleration, and bowel function return	Postoperative Misc endpoint	Adjuvant	31968595
Impact of perioperative enteral immunonutrition in patients with gastrointestinal cancer undergoing elective surgery: a randomized controlled trial	202	Nutritional status (weight loss and prealbumin) and functional capacities	Weight/Composition/Muscle	Adjuvant	Not found
Effect of Qihuang Decoction Combined with Enteral Nutrition on Postoperative Gastric Cancer of Nutrition and Immune Function	237	Blood markers of nutrition and immune function	Translational/Basic Science Endpoint	Adjuvant	32215032
Impact of hypocaloric and hyponitrogenic parenteral nutrition on clinical outcome in postoperative patients: A multi-center randomized controlled trial of 120 cases	120	Postoperative outcomes	Postoperative Misc endpoint	Adjuvant	17919374
Roles of clinical pharmacists in nutritional therapy for gastric cancer patients treated with neoadjuvant chemotherapy	60	Nutritional status and incidence of adverse reactions	Non-standard Quality of Life	Adjuvant	Not found
Effect of postoperative precision nutrition therapy on postoperative recovery for advanced gastric cancer after neoadjuvant chemotherapy	71	Postoperative nutritional status	Postoperative Misc endpoint	Adjuvant	Not found
Nutritional support of pediatric patients with cancer consuming an enteral formula with fructooligosaccharides	67	Stool microbiota (enterobacteria, bifidobacteria, lactobacillus, and clostridium perfringens in stool cultures)	Translational/Basic Science Endpoint	Adjuvant	Not found
Effect of oral nutritional supplementation on the post-discharge nutritional status and quality of life of gastrointestinal cancer patients after surgery: a multi-center study	114	Postoperative nutritional status and anthropometric measures	Weight/Composition/Muscle	Adjuvant	31464391
Improvement of constipation in leukemia patients undergoing chemotherapy using sweet potato	120	Constipation	Non-standard Quality of Life	Adjuvant	25881813

Supplementary Table 2

The following table describes the dietary interventions used in RCTs for cancer patients with assigned dietary categorization.

Title	Author	Dietary Intervention	Dietary Categorization	PubMed ID
Lack of effect of a high-fiber cereal supplement on the recurrence of colorectal adenomas	Alberts et al.	High fiber diet (13.5 g wheat bran fiber per day)	Fiber modified diet	10770890
Impact of Preoperative Acetaminophen and Carbohydrate Loading on Pain and Functional Status in Patients Undergoing Mohs Micrographic Surgery for Nonmelanoma Skin Cancers	Aleisa et al.	Preoperative carbohydrate loading and acetoaminophen	Energy modified diet	31725692
Oral nutritional supplement (ONS) reduced weight loss and optimised preoperative energy and nutrient intakes in colorectal cancer patients undergoing surgery	Ali et al.	Oral nutritional supplements	Nutrient modified diet	Not found
A Translational Randomized Trial of Perioperative Arginine Immunonutrition on Natural Killer Cell Function in Colorectal Cancer Surgery Patients	Angka et al.	Arginine enriched supplements	Nutrient modified diet	35913670
Vitamin D Supplementation and Survival in Metastatic Colorectal Cancer	Antunac et al.	Vitamin D supplementation	Nutrient modified diet	29533115
Effects of perioperative Eicosapentaenoic acid-enriched oral nutritional supplement on lean body mass after total gastrectomy for gastric cancer	Aoyama et al.	Eicosapentaenoic acid-enriched oral nutrition	Nutrient modified diet	30854113
Efficacy of ω-3 supplementation on nutritional status, skeletal muscle, and chemoradiotherapy toxicity in cervical cancer patients: A randomized, triple-blind, clinical trial conducted in a middle-income country	Aredes et al.	Omega-3 supplementation	Nutrient modified diet	Not found
Effects of a parallel-arm randomized controlled weight loss pilot study on biological and psychosocial parameters of overweight and obese breast cancer survivors	Arikawa et al.	Caloric deficit (1000 kcal deficit) and exercise intervention	Energy modified diet	28702218
Phase II prospective randomized trial of a low-fat diet with fish oil supplementation in men undergoing radical prostatectomy	Aronson et al.	Low-fat diet and fish oil supplementation	Energy modified diet	22027686
Randomized study to compare the compliance of two prescription of nutritional supplementation in oncology inpatients	Arribas et al.	Nutritional supplementation - 600 kcal and 25 g protein per day	Energy modified diet	Not found
The Impact of a Ketogenic Dietary Intervention on the Quality of Life of Stage II and III Cancer Patients: A Randomized Controlled Trial in the Caribbean	Augustus et al.	Modified ketogenic diet	Energy modified diet	32791011
Mediterranean-style dietary pattern improves cancer-related fatigue and quality of life in men with prostate cancer treated with androgen deprivation therapy: A pilot randomised control trial	Baguley et al.	Mediterranean diet	Mediterranean diet	35565100
The impact of a mediterranean-style dietary pattern on cancer-related fatigue and quality of life in men with prostate cancer treated with androgen deprivation therapy: A pilot study	Baguley et al.	Mediterranean diet	Mediterranean diet	Not found
Simple nutritional intervention in patients with advanced cancers of the gastrointestinal tract, non-small cell lung cancers or mesothelioma and weight loss receiving chemotherapy: a randomised controlled trial	Baldwin et al.	Nutritional supplementation and diet (additional 600 kcal per day)	Energy modified diet	21733143
The effect of perioperative immunonutrition on surgical complications and quality of life in patients with head and neck cancer	Basmisirli et al.	Immunonutrient modified entereal formula	Nutrition support	Not found
The effects of short-term fasting on quality of life and tolerance to chemotherapy in patients with breast and ovarian cancer: A randomized cross-over pilot study	Bauersfeld et al.	Short term fasting (max 350 kcal)	Energy modified diet	29699509
The use of a protein and energy dense eicosapentaenoic acid containing supplement for malignancy-related weight loss in children	Bayram et al.	Protein and energy dense eicosapentaenoic acid	Nutrient modified diet	19090549
Comparing diets for weight loss and improvement in biomarkers in men with prostate cancer on surveillance: A pilot study	Benson et al.	Plant based olive oil diet	Restrictive eating patterns	Not found
Influence of dietary factors on actinically-induced skin cancer	Black et al.	Low-fat diet	Energy modified diet	9920444
A double-blind phase III trial of immunomodulating nutritional formula during adjuvant chemoradiotherapy in head and neck cancer patients: IMPATOX	Boisselier et al.	Immunomodulating arginiene/omega-3 supplements	Nutrient modified diet	32936874
Effect of soy protein isolate supplementation on biochemical recurrence of prostate cancer after radical prostatectomy: A randomized trial	Bosland et al.	Soy protein isolate supplements	Energy modified diet	33764851
Modification of the response to chemotherapy of HER2 negative metastatic breast cancer by lipids of marine origin: A controlled, randomized, double blind dietary supplementation trial	Bougnoux et al.	Supplement long chain PUFA of marine origin	Energy modified diet	Not found
Nutritional advice in older patients at risk of malnutrition during treatment for chemotherapy: a two-year randomized controlled trial	Bourdel-Marchasson et al.	Diet counselling increasing calorie and protein intake	Energy modified diet	25265392
Glutamine supplementation in cancer patients receiving chemotherapy: A double-blind randomized study	Bozzetti et al.	Glutamine supplementation	Nutrient modified diet	9263281

Preoperative oral arginine and n-3 fatty acid supplementation improves the immunometabolic host response and outcome after colorectal resection for cancer	Braga et al.	Preoperative oral arginine and n3 fatty acid supplements	Nutrient modified diet	12464864
Immune and nutritional effects of early enteral nutrition after major abdominal operations	Braga et al.	Immunonnutrition enteral feeding supplementation	Nutrition support	8639722
Effect of fish oil on appetite and other symptoms in patients with advanced cancer and anorexia/cachexia: a double-blind, placebo-controlled study	Bruera et al.	Fish oil supplementation	Nutrient modified diet	12506181
Whey Protein Supplementation Improves Nutritional Status, Glutathione Levels, and Immune Function in Cancer Patients: A Randomized, Double-Blind Controlled Trial	Bumrungpert et al.	Whey protein isolates with zinc and selenium supplementation	Nutrient modified diet	29565716
Monitoring dietary change in a low-fat diet intervention study: advantages of using 24-hour dietary recalls vs food records	Buzzard et al.	Low-fat diet	Energy modified diet	Not found
The influence of low fat, low lactose diet on diarrhoea during pelvic radiotherapy	Bye et al.	Low-fat and low-lactose diet	Energy modified diet	7900512
The impact of preoperative immunonutrition and other nutrition models on tumor infiltrative lymphocytes in colorectal cancer patients	Caglayan et al.	Immunomodulating supplementation	Nutrient modified diet	23010614
A Whole-Food, Plant-Based (WFPB) dietary intervention to improve outcomes in patients with metastatic breast cancer	Campbell et al.	Whole food plant-based diet	Restrictive eating patterns	38045318
A Dietary Intervention for Recurrent Prostate Cancer After Definitive Primary Treatment: Results of a Randomized Pilot Trial	Camrody et al.	Dietary classes emphasizing a plant-based diet, fish, and vegetables	Nutrition counseling	18400281
Effect of Nutritional Supplementation Enriched with Eicosapentaenoic Acid on Inflammatory Profile of Patients With Oral Cavity Cancer in Antineoplastic Pretreatment: A Controlled and Randomized Clinical Trial	Carvalho et al.	Eicosapentaenoic acid supplementation	Nutrient modified diet	28128983
Pancreatic enzyme supplementation after gastrectomy for gastric cancer: a randomized controlled trial	Catarci et al.	Pancreatic enzyme supplementation	Nutrient modified diet	28804801
Social cognitive outcomes are associated with improvements in mobility performance following lifestyle intervention in prostate cancer patients undergoing androgen deprivation therapy	Chaplow et al.	Exercise and dietary intervention	Nutrition counseling	35085341
Role of enteral immunonutrition in patients with gastric carcinoma undergoing major surgery	Chen et al.	Enteral immunonutrition	Nutrient modified diet	15851366
Effects of Five-Step Nutritional Interventions Conducted by a Multidisciplinary Care Team on Gastroenteric Cancer Patients Undergoing Chemotherapy: A Randomized Clinical Trial	Chen et al.	Five step nutritional intervention on nutritional education	Nutrition counseling	35903847
A Phase 3 Randomized Trial of Nicotinamide for Skin-Cancer Chemoprevention	Chen et al.	500 mg of nicotinamide twice daily	Nutrient modified diet	26488693
The influence of low-carbohydrate diets on the metabolic response to androgen-deprivation therapy in prostate cancer	Chi et al.	Low-carbohydrate diet	Energy modified diet	33949711
Arginine, glutamine, and fish oil supplementation in cancer patients treated with concurrent chemoradiotherapy: A randomized control study	Chitapanarux et al.	Enteral arginine, glutamine, and fish oil supplements	Nutrient modified diet	31146957
Dietary fat reduction in postmenopausal women with primary breast cancer: Phase III Women's Intervention Nutrition Study (WINS)	Chlebowski et al.	Low-fat diet	Energy modified diet	Not found
Effects of Single Nucleotide Polymorphisms and Mediterranean Diet in Overweight or Obese Postmenopausal Women With Breast Cancer Receiving Adjuvant Hormone Therapy: A Pilot Randomized Controlled Trial	Cho et al.	Mediterranean diet	Mediterranean diet	35845810
Preoperative Immunonutrition in Liver Resection for Cancer: Results of the PROPILS Trial, a Multicenter Randomized Controlled Phase IV Trial	Ciacio et al.	Preoperative oral immunonutrition	Nutrient modified diet	Not found
Changes in dietary habits of women with breast cancer: preliminary results of a randomized controlled trial	Cioffi et al.	Counseling based on mediterranean diet	Mediterranean diet	Not found
A ketogenic diet reduces central obesity and serum insulin in women with ovarian or endometrial cancer	Cohen et al.	Ketogenic diet	Energy modified diet	30137481
Effect of nutritional counseling combined with oral nutritional supplements on clinical outcome of esophageal cancer patients under radiotherapy treatment	Cong et al.	Oral nutritional supplements	Nutrient modified diet	Not found
Impact of glutamine, eicosapntemacnioc acid, branched-chain amino acid supplements on nutritional status and treatment compliance of esophageal cancer patients on concurrent chemoradiotherapy and gastric cancer patients on chemotherapy	Cong et al.	Glutamine, arginine, and BCAA supplements	Nutrient modified diet	26080850
Effect of oral nutritional supplementation on nutritional status and quality of life in patients with gastric cancer after operation (23 cases RCT observations)	Cui et al.	Oral nutritional supplements	Nutrient modified diet	Not found
Enteral nutrition with supplemental arginine, RNA, and omega-3 fatty acids in patients after operation: Immunologic, metabolic, and clinical outcome	Daly et al.	Arginine, RNA, and omega-3 fatty acids	Nutrient modified diet	1377838
A Randomized Double-Blind Placebo-Controlled Trial of Fruit and Vegetable Concentrates on Intermediate Biomarkers in Head and Neck Cancer	Datta et al.	Fruit and vegetable concentrates	Nutrient modified diet	28102098

Fish oil supplementation and inflammatory response during neoadjuvant chemoradiation for rectal cancer: Results from a prospective, randomized, controlled trial	De Aguiar pastore silva et al.	Fish oil supplementation	Nutrient modified diet	Not found
Preoperative Fasting Abbreviation With Whey Protein Reduces the Occurrence of Postoperative Complications in Patients With Head and Neck Cancer: A Randomized Clinical Trial	De carvalho et al.	Whey protein	Energy modified diet	33373478
Fasting mimicking diet as an adjunct to neoadjuvant chemotherapy for breast cancer in the multicentre randomized phase 2 DIRECT trial	De groot et al.	Energy modified diet (plant-based, low calorie, low amino acid substitution)	Energy modified diet	32576828
A randomized clinical trial with oral immunonutrition (ω3-enhanced formula vs. arginine-enhanced formula) in ambulatory head and neck cancer patients	De luis et al.	Arginine enriched supplements	Nutrient modified diet	15802904
Fish oil supplementation during chemotherapy increases posterior time to tumor progression in colorectal cancer	De quadros carmargo et al.	Fish oil supplementation	Nutrient modified diet	26700096
Nutritional intervention contributes to the improvement of symptoms related to quality of life in breast cancer patients undergoing neoadjuvant chemotherapy: A randomized clinical trial	De souza et al.	Personalized diet plan	Nutrition counseling	Not found
Nutrition therapy in cachectic cancer patients. The Tight Caloric Control (TiCaCo) pilot trial	De waele et al.	Patient-specific dietary program coached by dietition	Nutrition counseling	Not found
Nutrition therapy promotes overall survival in cachectic cancer patients through a biophysical pathway: the ticaconco trial	De waele et al.	Intensive personalised dietary counseling	Nutrition counseling	Not found
Benefits of immunonutrition in patients with head and neck cancer receiving chemoradiation: A phase II randomized, double-blind study	Dechaphunkul et al.	Immunonutrient supplements	Nutrient modified diet	35007812
Results of a diet/exercise feasibility trial to prevent adverse body composition change in breast cancer patients on adjuvant chemotherapy	Demark-Wahnefried et al.	High fruit and vegetable, high calcium, low-fat diet along with exercise	Nutrition counseling	18501061
Flaxseed supplementation (not dietary fat restriction) reduces prostate cancer proliferation rates in men presurgery	Demark-Wahnefried et al.	Flaxseed supplementation	Nutrient modified diet	19064574
Presurgical weight loss affects tumour traits and circulating biomarkers in men with prostate cancer	Demark-Wahnefried et al.	Energy-restricted diet and exercise	Energy modified diet	28881355
Randomized trial of weight loss in primary breast cancer: Impact on body composition, circulating biomarkers and tumor characteristics	Demark-Wahnefried et al.	Counseling for resistance training, caloric restriction, and aerobic exercise	Nutrition counseling	31442303
Observation on application effect of TCM diet intervention in improving malnutrition in patients with end-stage liver cancer	Deng et al.	Traditional Chinese medicine dietary intervention	Restrictive eating patterns	Not found
Effects of a high dose, aglycone-rich soy extract on prostate-specific antigen and serum isoflavone concentrations in men with localized prostate cancer	Devere white et al.	High-dose isoflavone supplements	Nutrient modified diet	21058191
A Very-Low-Fat Vegan Diet Increases Intake of Protective Dietary Factors and Decreases Intake of Pathogenic Dietary Factors	Dewell et al.	Very low-fat vegan diet	Restrictive eating patterns	18237581
Effects of nutritional support in patients with colorectal cancer	Dintinjana et al.	Dietary counseling, oral nutrition supplements, and megestrol acetate	Nutrition counseling	18982745
Effect of adequate nutrition therapy in rectal carcinoma post-surgery patients	Djunet et al.	Adequate nutrition therapy	Nutrition counseling	Not found
A diet and exercise intervention during Chemotherapy for breast cancer	Djuric et al.	Nutrition counseling emphasizing low-fat, high-fruit diet and exercise	Energy modified diet	22238561
In a randomized trial in prostate cancer patients, dietary protein restriction modifies markers of leptin and insulin signaling in plasma extracellular vesicles	Eitan et al.	Protein restricted diet	Energy modified diet	Not found
Oral Nutritional Supplementation in Cancer Patients Who Were Receiving Chemo/Chemoradiation Therapy: A Multicenter, Randomized Phase II Study	Faccio et al.	Oral nutritional supplement (whey protein isolated, leucin, and zinc)	Nutrient modified diet	32363940
Reduced infections with perioperative immunonutrition in head and neck cancer: Exploratory results of a multicenter, prospective, randomized, double-blind study	Falewee et al.	Preoperative or perioperative immunonutrition	Nutrient modified diet	24182765
Perioperative nutritional support in patients undergoing hepatectomy for hepatocellular carcinoma	Fan et al.	BCAA, dextrose, and lipid emulsion supplementation	Nutrition support	7969324
Total parenteral alimentation with glutamine supplementation for the prevention of common complications in patients undergoing hematopoietic stem cell transplantation	Fang et al.	Glutamine enriched TPN	Nutrient modified diet	Not found

Effect of conjugated linoleic acid supplementation on quality of life in rectal cancer patients undergoing preoperative chemoradiotherapy	Faramarzi et al.	Conjugated linoleic acid supplementation	Nutrient modified diet	28523042
Effects of ω-3 supplementation on the nutritional status, immune, and inflammatory profiles of gastric cancer patients: A randomized controlled trial	Feijo et al.	Omega-3 supplementation	Nutrient modified diet	30710885
Effects of a nutrition intervention on acute and late bowel symptoms and health-related quality of life up to 24 months post radiotherapy in patients with prostate cancer: a multicentre randomised controlled trial	Forslund et al.	Dietary counseling promoting high soluble fiber, low-lactose diet	Fiber modified diet	31758324
Dynamic metabolic response of prostate cancer patients treated with ADT and low carb diet	Freedland et al.	Low-carb diet	Energy modified diet	Not found
A Randomized Controlled Trial of a 6-Month Low-Carbohydrate Intervention on Disease Progression in Men with Recurrent Prostate Cancer: Carbohydrate and Prostate Study 2 (CAPS2)	Freedland et al.	Low-carb diet	Energy modified diet	33712498
Effect of postoperative diet nursing with patient involved on nutritional status of the patients with rectal cancer	Gan et al.	Postoperative diet nursing	Nutrition counseling	Not found
Effect of Targeted Nutritional Intervention on Intestinal Flora, Defecation Function, and Postoperative Complications in Patients Who Underwent Radical Resection of Rectal Carcinoma	Gao et al.	Targeted nutritional intervention with probiotic supplementation	Nutrient modified diet	Not found
Randomized comparison of cooked and noncooked diets in patients undergoing remission induction therapy for acute myeloid leukemia	Gardner et al.	Neutropenic diet	Restrictive eating patterns	18955453
Effect of Standardized Nutritional Intervention in Patients with Nasopharyngeal Carcinoma Receiving Radiotherapy Complicated with Diabetes Mellitus	Ge et al.	Nutritional intervention via online for weight and blood glucose control	Nutrition counseling	35756497
Immune Nutrition in Head and Neck Cancer. A Double Blind Randomised Controlled Trial of Perioperative Immune Enhancing Feeds in Patients with Advanced Head and Neck Cancer	Ghosh et al.	Immune enhancing feeds	Nutrition support	Not found
A randomized controlled trial of preoperative oral supplementation with a specialized diet in patients with gastrointestinal cancer	Gianotti et al.	Supplemented liquid diet	Nutrient modified diet	12055582
Route and composition of postoperative nutritional support: Impact on immune-metabolic response and postoperative outcome	Gianotti et al.	Immunonutrition	Nutrition support	Not found
Short-term preoperative supplementation of an immunoenriched diet does not improve clinical outcome in well-nourished patients undergoing abdominal cancer surgery	Giger pabst et al.	Immuno enriched formula	Nutrient modified diet	Not found
Evaluation of perioperative nutritional therapy in patients with gastrointestinal tract neoplasms	Gomez sanchez et al.	Perioperative immune enhancing formula	Nutrient modified diet	22072355
Efficacy evaluation of an oral powder supplement enriched with eicosapentaenoic acid in cancer patients	Gomez candela	Eicosapentaenoic acid supplementation	Nutrient modified diet	22411387
A combination of tomato and soy products for men with recurring prostate cancer and rising prostate specific antigen	Grainger et al.	Diet rich in tomato products and soy protein supplements	Nutrient modified diet	18444145
Effects of a dietary intervention to increase omega-3 intake compared to dutasteride in men with low-risk prostate cancer under active surveillance: Preliminary results on fatty acid intake and fatty acid profiles of red blood cells and prostate tissue	Guertin et al.	Dietary intervention aiming to increase omega-3 intake	Nutrient modified diet	Not found
The effect of immunonutrition on tumor infiltrative t lymphocytes and regulatory t cells in rectal tumor patients receiving neoadjuvant chemoradiotherapy: a prospective randomized clinical study	Gul et al.	Immunonutrition	Nutrient modified diet	36326418
Effects of emotional intervention and Chinese medicated diet in improving the quality of life in patients with liver cancer after chemotherapy	Guo et al.	Chinese medicated diet	Restrictive eating patterns	Not found
A Pilot Randomised Controlled Trial Examining the Benefit of a Neutropenic Diet for Children Undergoing Cancer Treatment	Gupta et al.	Neutropenic diet	Restrictive eating patterns	35225115
Assessment of Nutritional Status in Children with Cancer and Effectiveness of Oral Nutritional Supplements	Gurlek et al.	Hypercaloric oral supplements	Nutrient modified diet	26418028
Application of Glutamine-enriched nutrition therapy in childhood acute lymphoblastic leukemia	Han et al.	Glutamine enriched nutrition	Nutrient modified diet	27401338
Prospective randomized investigation implementing immunonutritional therapy using a nutritional supplement with a high blend ratio of ω-3 fatty acids during the perioperative period for head and neck carcinomas	Hanai et al.	Eicosapentaenoic acid supplementation	Nutrient modified diet	29420749
Miami NICE trial: Nutritional support for patients incurring chemotherapy side effects	Hansra et al.	Counseling for dietary strategies to mitigate side effects	Nutrition counseling	Not found
Randomized controlled clinical trial assessing the effects of oral nutritional supplements in postoperative gastric cancer patients	Hatao et al.	Oral nutritional supplements	Nutrient modified diet	27807617
Change in women's diet and body mass following intensive intervention for early-stage breast cancer	Herbert et al.	Nutrition education program	Nutrition counseling	11320947

Immunonutrition in elective gastrointestinal surgery patients	Helminen et al.	Perioperative immunonutrition	Nutrient modified diet	17461312
Dietary intervention as adjuvant therapy in breast cancer patients - a feasibility study	Holm et al.	Dietary counseling to reduce fat intake	Nutrition counseling	2265258
A double-blind randomized controlled trial of the effects of eicosapentaenoic acid supplementation on muscle inflammation and physical function in patients undergoing colorectal cancer resection	Hossain et al.	Eicosapentaenoic acid supplementation	Nutrient modified diet	31648815
Efficacy of Omega Fatty Acid Supplementation on mRNA Expression Level of Tumor Necrosis Factor Alpha in Patients with Gastric Adenocarcinoma	Hosseinzadeh et al.	Polyunsaturated fatty acid supplements	Nutrient modified diet	27170003
A prospective randomized controlled trial on the value of prophylactic oral nutritional supplementation in locally advanced nasopharyngeal carcinoma patients receiving chemo-radiotherapy	Huang et al.	Oral nutritional supplements	Nutrient modified diet	33032180
The impact of nutrition support on body composition in cancer outpatients receiving radiotherapy	Isenring et al.	Counseling following the American Dietetic Association	Nutrition counseling	15226773
Oral nutritional support can shorten the duration of parenteral hydration in end-of-life cancer patients: A randomized controlled trial	Ishiki et al.	Oral nutritional supplements	Nutrient modified diet	25437180
Efficacy of EPA-enriched supplement compared with standard formula on body weight changes in malnourished patients with head and neck cancer undergone surgery: a randomized study	Jantharapattana et al.	Eicosapentaenoic acid supplementation	Nutrient modified diet	31647147
An eicosapentaenoic acid supplement versus megestrol acetate versus both for patients with cancer-associated wasting: A North Central Cancer Treatment Group and National Cancer Institute of Canada collaborative effort	Jatoi et al.	Eicosapentaenoic acid supplementation	Nutrient modified diet	15197210
Structured triglycerides were well tolerated and induced after hepatectomy in patients with hepatocarcinoma	Jia et al.	Nutrition with structured triglycerides	Nutrient modified diet	Not found
Effect of glutamine's nutrition support on the postoperative nutrition and immune function in malignant tumor of gynecology patients	Jiang et al.	Enteral immunonutrition	Nutrition support	25016261
Benefits of Oral Nutritional Supplements in Patients with Locally Advanced Nasopharyngeal Cancer during Concurrent Chemoradiotherapy: An Exploratory Prospective Randomized Trial	Jiang et al.	Oral nutritional supplements	Nutrient modified diet	30633580
Vitamin d supplementation and disease-free survival in stage ii melanoma: A randomized placebo controlled trial	Johanson et al.	Vitamin D supplementation	Nutrient modified diet	34199802
Muscle mass, strength, and index in various models of preoperative immunonutrition in invasive gastric cancer patients	Jurczuk et al.	Immunonutrition	Nutrient modified diet	Not found
Preoperative nutritional support in cancer patients with no clinical signs of malnutrition—prospective randomized controlled trial	Kabata et al.	Oral nutritional supplements	Nutrient modified diet	25091056
Phagocytic activity of blood platelets in various models of preoperative oral immunonutrition in invasive cancer patients	Kamocki et al.	Immunonutrition	Nutrient modified diet	Not found
Lymphocyte subpopulation in a model of preoperative oral and parenteral glutamine-based immunonutrition in patients with invasive gastric cancer	Kamocki et al.	Immunonutrition	Nutrient modified diet	Not found
Efficacy of perioperative immunonutrition in esophageal cancer patients undergoing esophagectomy	Kanekiyo et al.	Perioperative immunonutrition	Nutrient modified diet	30468936
A Prospective Randomized Controlled Trial to Study the Impact of a Nutrition-Sensitive Intervention on Adult Women with Cancer Cachexia Undergoing Palliative Care in India	Kapoor et al.	Immunomodulating supplementation with Improved Atta	Nutrient modified diet	Not found
Randomized study of prevention of gastrointestinal toxicities by nutritional support using an amino acid-rich elemental diet during chemotherapy in patients with esophageal cancer (KDOG 1101)	Katada et al.	Amino acid rich elemental diet supplementation	Nutrient modified diet	33009977
Effects of 4 weeks of Lactobacillus plantarum 299v supplementation on nutritional status, enteral nutrition tolerance, and quality of life in cancer patients receiving home enteral nutrition - A double-blind, randomized, and placebo-controlled trial	Kazmierzak et al.	Lactobacillus supplementation	Nutrient modified diet	33015813
Early postoperative enteral nutrition with arginine-ω-3 fatty acids and ribonucleic acid-supplemented diet versus placebo in cancer patients: An immunologic evaluation of Impact®	Kemen et al.	Arginine, RNA, and omega-3 fatty acids	Nutrient modified diet	7536138
Early enteral feeding in postsurgical cancer patients. Fish oil structured lipid-based polymeric formula versus a standard polymeric formula	Kenler et al.	Fish oil supplementation	Nutrient modified diet	8604913
Effects of Ketogenic metabolic therapy on patients with breast cancer: A randomized controlled clinical trial	Khodabakhshi et al.	Ketogenic diet	Energy modified diet	Not found
A Randomized Clinical Trial of Preoperative Administration of Branched-Chain Amino Acids to Prevent Postoperative Ascites in Patients with Liver Resection for Hepatocellular Carcinoma	Kikuchi et al.	BCAA supplementation	Nutrient modified diet	27338747
The impact of nutritional support on treatmentrelated complications, qol and survival in lung cancer patients undergoing radiotherapy: A randomized, controlled study	Kilic et al.	Immunoenriched oral nutritional supplements	Nutrient modified diet	Not found

The effects of patient participation-based dietary intervention on nutritional and functional status for patients with gastrectomy: A randomized controlled trial	Kim et al.	Patient participation dietary counseling to increase energy intake	Nutrition counseling	23632471
The Effect of Nutrition Intervention with Oral Nutritional Supplements on Pancreatic and Bile Duct Cancer Patients Undergoing Chemotherapy	Kim et al.	Oral nutritional supplements	Nutrient modified diet	31121926
The effect of preoperative nutritional supports on patients with gastrointestinal cancer: Prospective randomized study	Kirkil et al.	Preoperative nutritional supports	Nutrient modified diet	22260826
The Effects of a Mediterranean Diet Intervention on Cancer-Related Fatigue for Patients Undergoing Chemotherapy: A Pilot Randomized Controlled Trial	Kleckner et al.	Mediterranean diet	Mediterranean diet	36077737
The impact of immunostimulating nutrition on infectious complications after upper gastrointestinal surgery: A prospective, randomized, clinical trial	Klek et al.	Immunomodulating nutrition	Nutrient modified diet	18650630
Standard and immunomodulating enteral nutrition in patients after extended gastrointestinal surgery--a prospective, randomized, controlled clinical trial	Klek et al.	Enteral nutrition with immunostimulating diet	Nutrition support	18571296
L-Carnitine-supplementation in advanced pancreatic cancer (CARPAN) - A randomized multicentre trial	Kraft et al.	L-carnitine supplementation	Nutrient modified diet	22824168
Dietary intervention in prostate cancer patients: PSA response in a randomized double-blind placebo-controlled study	Kranse et al.	Supplementation of plant estrogens, antioxidants, carotenoids, and selenium	Nutrient modified diet	Not found
Long term improved quality of life by a 2-week group physical and educational intervention shortly after breast cancer chemotherapy completion. Results of the 'Programme of Accompanying women after breast Cancer treatment completion in Thermal resorts' (PACThe) randomised clinical trial of 251 patients	Kwiatkowski et al.	2-week session of physical training, dietary education, and physiotherapy	Nutrition counseling	23352440
Influence of pre-operative oral carbohydrate loading vs. standard fasting on tumor proliferation and clinical outcome in breast cancer patients ─ a randomized trial	Lende, T. H., et al.	Preoperative oral carbohydrate loading	Energy modified diet	31703648
Nursing Effect and Prognosis of Perioperative Nutritional Support Therapy for Severe Malnutrition Patients with Colorectal Cancer	Li, C. et al.	Perioperative enteral nutritional support	Nutrition support	Not found
Early enteral immunonutrition support protects the cellular and humoral immune functions of patients with pancreatic cancer after chemotherapy	Li, C., et al.	Immunomodulating entereal nutrition	Nutrition support	Not found
The effect of low-nitrogen and low-calorie parenteral nutrition combined with enteral nutrition on inflammatory cytokines and immune functions in patients with gastric cancer: a double blind placebo trial	Li, J. H., et al.	Low-nitrogen and low-calorie parenteral nutrition with enteral nutrition	Nutrition support	25967707
A randomized nutrition counseling intervention in pediatric leukemia patients receiving steroids results in reduced caloric intake	Li, R., et al.	Dietary counsel following the American Academy of Pediatrics recomendation	Nutrition counseling	27615542
Enteral immunonutrition versus enteral nutrition for patients undergoing esophagectomy: a double-blinded randomized controlled trial	Li, X., et al.	Enteral immunonutrition	Nutrition support	Not found
Feasibility of a low-fat/high-fiber diet intervention with soy supplementation in prostate cancer patients after prostatectomy	Li, Z., et al.	Low-fat fiber modified diet	Fiber modified diet	17392697
A multidisciplinary team approach for nutritional interventions conducted by specialist nurses in patients with advanced colorectal cancer undergoing chemotherapy: A clinical trial	Lin, J. et al.	Individual recipes developed by a team of professionals and the patient	Nutrition counseling	28658162
Effects of Zinc Supplementation on Clinical Outcomes in Patients Receiving Radiotherapy for Head and Neck Cancers: A Double-Blinded Randomized Study	Lin, L. et al.	Zinc supplementation	Nutrient modified diet	17980503
Effect of Psychological Intervention Combined with Dietary Guidance on Quality of Life and Long-Term Efficacy of Bushen Quyu Decoction in Treatment of Patients with Advanced Ovarian Cancer	Liu, D. et al.	Psychological counseling to maintain a light diet	Nutrition counseling	34733335
An enhanced nutritional support pathway including extended preoperative and home enteral nutrition is safe, feasible, and may benefit patients undergiong enhanced recovery after esophagectomy: A pilot randomized clinical trial	Liu, K. et al.	Standard nutrional support	Nutrition support	Not found
A Randomized Study on the Effect of Metformin Combined with Intensive-Exercise Diet Therapy on Glucose and Lipid Metabolism and Islet Function in Patients with Renal Cell Carcinoma and Diabetes	Liu, Y. et al.	Counseling for an energy modified diet and excercise	Nutrition counseling	Not found
Early postoperative jejunostomy feeding with an immune modulating diet in patients undergoing resectional surgery for upper gastrointestinal cancer: a prospective, randomized, controlled, double-blind study	Lobo et al	Postoperative immunomodulating nutrition	Nutrient modified diet	Not found
Feasibility and efficacy of progressive resistance training and dietary supplements in radiotherapy treated head and neck cancer patients-the DAHANCA 25A study	Lonbro, S. et al.	Protein and creatinine supplementation before excercise	Nutrient modified diet	23190359
Analysis of the Effect of Exercise Combined with Diet Intervention on Postoperative Quality of Life of Breast Cancer Patients	Lu, L.	High protein, high energy, and high vitamin supplementation	Nutrient modified diet	35669367

Palliative Nutritional Intervention in Addition to Cyclooxygenase and Erythropoietin Treatment for Patients with Malignant Disease: Effects on Survival, Metabolism, and Function: A Randomized Prospective Study	Lundholm, K. et al.	Counseling with oral and parenteral supplements (450–600 kcal per day)	Nutrition support	15112279
The effect of zinc sulfate in the prevention of high-dose chemotherapy-induced mucositis: a double-blind, randomized, placebo-controlled study	Mansouri, A. et al.	Zinc supplementation	Nutrient modified diet	21692101
Immunonutrition to improve the quality of life of upper gastrointestinal cancer patients undergoing neoadjuvant treatment prior to surgery (NEOIMMUNE): Double blind randomized controlled multi-center clinical trial	Markar, S. et al.	Immuno-nutrients supplementation	Nutrient modified diet	Not found
Ketogenic diets as an adjuvant therapy for glioblastoma (KEATING): a randomized, mixed methods, feasibility study	Martin-McGill, K. J.	Ketogenic Diet	Energy modified diet	Not found
An exercise and nutrition intervention for ovarian cancer patients during and after first-line chemotherapy (BENITA study): A randomized controlled pilot trial	Maurer, T. et al.	12-month exercise and increasing protein and calorie intake	Nutrition counseling	35197344
The effect of nutritional supplements on food intake in patients undergoing radiotherapy	McCarthy, D. et al.	Liquid nutritional supplement	Nutrient modified diet	10382188
Is supplementation with elemental diet feasible in patients undergoing pelvic radiotherapy?	McGough, C. et al.	Elemental nutritional supplement	Nutrient modified diet	16289498
Effect of preoperative immunonutrition in patients undergoing hepatectomy; a randomized controlled trial	Mikagi, K. et al.	Immunonutrition supplement	Nutrient modified diet	30741015
Efficacy of prolonged elemental diet therapy after pancreaticoduodenectomy for pancreatic ductal adenocarcinoma: A pilot prospective randomized trial (UMIN000004108)	Mori, R. et al.	Elemental nutritional supplement	Nutrient modified diet	31677701
Effects of medical nutrition therapy compared with general nutritional advice on nutritional status and nutrition-related complications in esophageal cancer patients receiving concurrent chemoradiation: A randomized controlled trial	Movahed, S. et al.	Individualized nutritional therapy	Nutrition counseling	Not found
Perioperative standard oral nutrition supplements versus immunonutrition in patients undergoing colorectal resection in an Enhanced Recovery (ERAS) protocol	Moya, P. et al.	Immunonutrition	Nutrient modified diet	Not found
Multicenter, phase III trial comparing selenium supplementation with observation in gynecologic radiation oncology: Follow-up analysis of the survival data 6 years after cessation of randomization	Muecke, R. et al.	Selenium supplementation	Nutrient modified diet	25015649
Effects of dietary supplements of pediasure and carnitine on the anthropometric indices in children with acute lymphoblastic leukemia under chemotherapy, in Afzalipour Hospital, Kerman, Iran	Naderi, A. et al.	Supplementation with Pediasure and carnitine	Nutrient modified diet	Not found
Impact of nutritional supplementation on treatment delay and morbidity in patients with head and neck tumors treated with irradiation	Nayel, H. et al.	Nutrition supplementation	Nutrient modified diet	1562782
Parenteral nutrition effects of Omega-3 fatty acids on C-reactive protein, high-density lipoprotein, lymphocyte characteristics and the treatment of critically ill patients	Ni, C. et al.	Omega-3 fish oil fat emulsion supplementation	Nutrient modified diet	32538747
Effects of branched-chain amino acid supplementation after radiofrequency ablation for hepatocellular carcinoma: A randomized trial	Nojiri, S. et al.	BCAA supplementation	Nutrient modified diet	27908546
Prostatic concentration of soy isoflavone exceeds serum after dietary supplementation	Oelrich, B. et al.	Daily soy supplements	Nutrient modified diet	
L-Alanin-L-Glutamine supplementation improves the outcome after colorectal surgery for cancer	Oguz, M. et al.	L-Alanin-L-Glutamine supplementation	Nutrient modified diet	17573745
Oral supplementation with carbohydrate- and branched-chain amino acid-enriched nutrients improves postoperative quality of life in patients undergoing hepatic resection	Okabayashi, T. et al.	Supplementation with BCAA-enriched nutrient	Nutrient modified diet	20852905
Attenuation of the systemic inflammatory response and infectious complications after gastrectomy with preoperative oral arginine and ω-3 fatty acids supplemented immunonutrition	Okamoto, Y. et al.	Fatty acids supplemented immunonutrition	Nutrient modified diet	19629583
Nutritional behaviour and quality of life during oncological polychemotherapy: Results of a prospective study on the efficacy of oral nutrition therapy in patients with acute leukaemia	Ollenschlager, G. et al.	Education and daily dietician visits to increase protein and calorie intake	Nutrition counseling	1425861
Nutritional supplementation with an immuneenhancing formula in the patients with esophageal cancer	Panova, N. et al.	Enteral immune-enhancing nutrition supplementation	Nutrient modified diet	Not found
Dramatic dietary fat reduction is feasible for breast cancer patients: Results of the randomised study, WINS (UK) - stage 1	Parry, B. M. et al.	Dietary counseling to halve their reported fat intake	Energy modified diet	21868187
The Men's Eating and Living (MEAL) Study: A Cancer and Leukemia Group B Pilot Trial of Dietary Intervention for the Treatment of Prostate Cancer	Parsons, J. K. et al.	Telephone-based counseling to increase vegetables, whole grains, and beans/legumes	Nutrition counseling	Not found
A randomized pilot trial of dietary modification for the chemoprevention of noninvasive bladder cancer: The Dietary Intervention in Bladder Cancer Study	Parsons, J. K. et al.	Telephone and Skype dietary counseling	Nutrition counseling	23867158

Effect of a Behavioral Intervention to Increase Vegetable Consumption on Cancer Progression Among Men With Early-Stage Prostate Cancer: The MEAL Randomized Clinical Trial	Parsons, J. K. et al.	Telephone-based counseling	Nutrition counseling	32586177
A randomized study of nutritional support in patients with colorectal and gastric cancer	Persson, C. R. et al.	Individualized nutritional support	Nutrition counseling	12235650
Effects of a dietary intervention on acute gastrointestinal side effects and other aspects of health-related quality of life: A randomized controlled trial in prostate cancer patients undergoing radiotherapy	Pettersson, A. et al.	Low soluble fiber and lactose diet	Nutrition counseling	22633817
Shrinking preoperative fast time with maltodextrin and protein hydrolysate in gastrointestinal resections due to cancer	Pexe-Machado, P. A. et al.	Hydrolyzed protein to a carbohydrate-based nutrition supplementation	Nutrient modified diet	23759267
Influence of a diet very high in vegetables, fruit, and fiber and low in fat on prognosis following treatment for breast cancer: the Women's Healthy Eating and Living (WHEL) randomized trial	Pierce, J. P. et al.	High in vegetables, fruit, and fiber and low in fat	Nutrient modified diet	17635889
Long-term oral branched chain amino acids in patients undergoing chemoembolization for hepatocellular carcinoma: A randomized trial	Poon, R. T. P. et al.	BCAA suplementation	Nutrient modified diet	15043519
Nutritional counseling in patients with head, neck and esophagus cancer in (chemo)radiotherapy	Pootz. S. et al.	Nutrional counseling on education for nutritional status	Nutrition counseling	Not found
Randomized trial of the effects of individual nutritional counseling in cancer patients	Poulsen, G. M. et al.	Individualized nutrition counseling	Nutrition counseling	24269077
Efficacy of ready-to-use therapeutic food in malnourished children with cancer: Results of a randomized, open-label phase 3 trial	Prasad, M. et al.	Ready-to-use therapeutic food supplementation	Nutrient modified diet	34212474
Postoperative early enteral nutrition therapy with long peptide mixture for gastric cancer patients: A clinical control study	Pu, J. et al.	Early enteral peptide-based feeding	Nutrition support	Not found
Effect of whole-course nutrition management on patients with esophageal cancer undergoing concurrent chemoradiotherapy: A randomized control trial	Qiu, Y. et al.	Nutrition support team	Nutrition counseling	31526964
Neutropenic versus regular diet for acute leukaemia induction chemotherapy: randomised controlled trial	Radhakrishnan, V. et al.	Neutropenic diet	Restrictive eating patterns	35803707
Association between amino acids, biomarkers of prostate cancer and inflammation in norwegian prostate cancer patients	Rangel Huerta, O. D. et al.	30 mg lycopene per day supplementation	Nutrient modified diet	Not found
Individualized nutrition intervention is of major benefit to colorectal cancer patients: Long-term follow-up of a randomized controlled trial of nutritional therapy	Ravasco, P. et al.	Individualized nutritional counseling	Nutrition counseling	23134880
Effect of a specific supplement enriched with n-3 polyunsaturated fatty acids on markers of inflammation, oxidativestress and metabolic status of ear, nose and throat cancer patients	Roca-Rodríguez, M. M. et al.	N-3 polyunsaturated fatty acids, fiber, and protein supplementation	Nutrient modified diet	Not found
Effect of Early Peripheral Parenteral Nutrition Support in an Enhanced Recovery Program for Colorectal Cancer Surgery: A Randomized Open Trial	Sanchez-Guillien	Perioperative peripheral nutrition of PeriOliclimonel	Nutrition support	Not found
Effects of an oral nutritional supplement containing eicosapentaenoic acid on nutritional and clinical outcomes in patients with advanced non-small cell lung cancer: randomised trial	Sánchez-Lara, K. et al.	Eicosapentaenoic acid supplementation	Nutrient modified diet	24746976
Effects of a Plant-Based High-Protein Diet on Fatigue in Breast Cancer Patients Undergoing Adjuvant Chemotherapy–a Randomized Controlled Trial	Sathiaraj, E. et al.	Plant-based high-protein diet	Restrictive eating patterns	36546552
Randomized, double-blind, controlled study of glycyl-glutamine-dipeptide in the parenteral nutrition of patients with acute leukemia undergoing intensive chemotherapy	Scheid, C. et al.	Parenteral support with glycyl-glutamine-dipeptide nutrition	Nutrition support	14990264
Randomized, double-blind, placebo-controlled crossover study in men with prostate cancer and rising PSA: Effectiveness of a dietary supplement	Schröder, F. H. et al.	Soy-based dietary supplement	Nutrient modified diet	16263208
Effects of an exercise and hypocaloric healthy eating program on biomarkers associated with long-term prognosis after early-stage breast cancer: A randomized controlled trial	Scott, E. et al.	Hypocaloric healthy eating	Energy modified diet	23184120
Effect of a Perioperative Nutritional Supplementation with Oral Impact® in Patients undergoing Hepatic Surgery for Liver Cancer: A Prospective, Placebo-Controlled, Randomized, Double-Blind Study	Seguin, P. et al.	Perioperative nutritional supplementation	Nutrient modified diet	27007018
Preoperative oral supplementation with long-chain Ω-3 fatty acids beneficially alters phospholipid fatty acid patterns in liver, gut mucosa, and tumor tissue	Senkal, M. et al.	Oral supplementation with long-chain omega-3 fatty acids	Nutrient modified diet	15961678
Comprehensive Diet and Exercise Intervention During Radiation Therapy Can Improve Metabolic and Inflammatory Markers in Breast Cancer Patients	Senthilkumar, G. et al.	Dietary counseling sessions	Nutrition counseling	Not found

To compare outcome of Intensive nutritional support with standard practise in head ands neck cancer	Shahid, T. et al.	Nutrition counseling following the American Dietetic Association	Nutrition counseling	Not found
Chinese food therapy combined with chemotherapy in the treatment of advanced ovarian cancer based on	Shang, C. L. et al.	Traditional Chinese Medicinal dietary supplements	Nutrient modified diet	Not found
Randomized controlled trial comparing a low-fat diet with a weight-reduction diet in breast cancer-related lymphedema	Shaw, C. et al.	Hypocaloric and low-fat diet	Energy modified diet	17393377
Application of the Diet and Nutrition Management System in the Nutrition Management of Tumor Patients Treated with Chemotherapy	She, G. et al.	Counseling following nutritional management system	Nutrition counseling	Not found
Influence of nutritional support on tolerability and results of treatment in patients with newly diagnosed hemoblastoses received program chemotherapy	Shen, N. P. et al.	Protein supplement for underweight patients	Energy modified diet	Not found
Effect of early nutritional support on the clinical efficacy of gastrointestinal cancer patients	Shen, X. J. et al.	Enteral nutrition in the postoperative treatment	Nutrition support	Not found
Phase II randomized controlled trial (RCT) of medical intensive nutrition therapy (MINT) to improve chemotherapy (CT) tolerability in malnourished patients with solid tumor malignancies	Shusterman, M. et al.	Counseling of medical intensive nutrition therapy	Nutrition counseling	Not found
Potential benefits of early nutritional intervention in adults with upper gastrointestinal cancer: a pilot randomised trial	Silvers, M. A. et al.	Telehealth nutritional counseling	Nutrition counseling	Not found
Anorexia-cachexia syndrome in cancer patients: Pathogenetic aspects and treatment options	Snegovoy, A. V. et al.	Enteral nutritional support	Nutrition support	Not found
Reduced postoperative infections with an immune-enhancing nutritional supplement	Snyderman, C. H. et al.	Immune-enhancing nutritional supplement	Nutrient modified diet	10369282
Randomised controlled trial of one week strict low-iodine diet versus one week non-specified low iodine diet in differentiated thyroid carcinoma	Sohaimi, W. F. W. et al.	Low-iodine diet	Restrictive eating patterns	Not found
Rapid Incorporation of ω-3 Fatty Acids into Colonic Tissue after Oral Supplementation in Patients with Colorectal Cancer: A Randomized, Placebo-Controlled Intervention Trial	Sorensen, L. S. et al.	ω-3 fatty acid supplementation	Nutrient modified diet	23788002
Effect of a leucine-rich supplement in combination with nutrition and physical exercise in advanced cancer patients: A randomized controlled intervention trial	Storck, L. J. et al.	Leucine-rich supplementation	Nutrient modified diet	32340904
Effect of targeted nutrition intervention on defecation and postoperative complications in patients undergoing radical resection for rectal cancer	Sun, Q. Y. et al.	In-patient management of care by a dietitian/nutritionist	Nutrition counseling	Not found
A randomized, double blind comparative study of prophylactic parenteral nutritional support with or without glutamine in autologous stem cell transplantation for hematological malignancies - Three years' follow-up	Sykorova, A. et al.	Glutamine-enriched parenteral nutrition	Nutrition support	16284692
Randomized Controlled Trial Investigating Perioperative Immunonutrition for Patients Undergoing Cytoreductive Surgery (CRS) and Hyperthermic Intraperitoneal Chemotherapy (HIPEC)	Tan, G. H. C. et al.	Immunonutrition	Nutrient modified diet	36394687
Effect of different nutritional support modes on humoral immunity and outcomes after esophagectomy	Tan, T. C. et al.	Mixed enteral nutrition or enteral nutrition and parenteral nutrition	Nutrition support	Not found
Evolution of inflammatory markers in patients with advanced head and neck carcinoma with prophylactic percutaneous gastrostomy	Tapia, M. J. et al.	Nutritional therapy vs. prophylactic gastrostomy	Nutrition counseling	Not found
Preoperative nutritional support for patients undergoing elective colorectal cancer surgery - does it really work?	Tesar, M. et al.	Preoperative oral nutritional supplements	Nutrient modified diet	35258042
A double blind, placebo controlled randomised trial (RCT) evaluating the effect of a polyphenol rich whole food supplement on PSA progression in men	Thomas, R. et al.	Oral supplement of pomegranate seed, green tea, broccoli, and turmeric	Nutrient modified diet	Not found
Longitudinal changes in body weight and body composition among women previously treated for breast cancer consuming a high-vegetable, fruit and fiber, low-fat diet	Thomson et al.	Diet high in fruit,vegetables and fiber, and low in fat	Restrictive eating patterns	15309460
beta-carotene supplementation in patients radically treated for stage I-II head and neck cancer: results of a randomized trial	Toma et al.	Beta carotene supplement	Nutrient modified diet	14534715
Transcriptional changes in prostate of men on active surveillance after a 12-mo glucoraphanin-rich broccoli intervention-results from the Effect of Sulforaphane on Prostate CAncer PrEvention (ESCAPE) randomized controlled trial	Traka et al.	Supplement of broccoli soup	Nutrient modified diet	30982861
Oral administration of the amino acids cystine and theanine attenuates the adverse events of S-1 adjuvant chemotherapy in gastrointestinal cancer patients	Tsuchiya et al.	Cystine and theanine supplementation	Nutrient modified diet	27306219
Perioperative immunonutrition in well-nourished patients undergoing surgery for head and neck cancer: Evaluation of inflammatory and immunologic outcomes	Turnock et al.	Immunonutrition supplementation	Nutrient modified diet	23571650

Glutamine-enriched nutrition does not reduce mucosal morbidity or complications after stem-cell transplantation for childhood malignancies: A prospective randomized study	Uderzo et al.	Parenteral supplementation with glutamine	Nutrition support	21499196
Influence of a nutritional intervention on dietary intake and quality of life in cancer patients: A randomized controlled trial	Uster et al.	Nutritional supplements and counseling for energy and protein requirement	Nutrition counseling	24103511
Effects of nutrition and physical exercise intervention in palliative cancer patients: A randomized controlled trial	Uster et al.	Nutritional counseling and exercise to meet protein requirements	Nutrition counseling	28651827
Calorie restriction and synbiotics effect on quality of life and edema reduction in breast cancer-related lymphedema, a clinical trial	Vafa et al.	Calorie restricted diet plus colony forming unit synbiotic supplement	Energy modified diet	32898787
Randomized phase 2 study of gemcitabine and cisplatin with or without vitamin supplementation in patients with advanced esophagogastric cancer	Van Zweeden et al.	Vitamin B12 and folic acid supplementation	Nutrient modified diet	29696360
Immunonutrition improves functional capacities in head and neck and esophageal cancer patients undergoing radiochemotherapy: A randomized clinical trial	Vasson et al.	Immunomodulating supplementation	Nutrient modified diet	23849811
Preventing weight gain during adjuvant chemotherapy for breast cancer: A dietary intervention study	Villarini et al.	Mediterranean diet	Mediterranean diet	22869285
ERGO2: A prospective randomized trial of a 9-day schedule of calorically restricted ketogenic diet and fasting or standard diet in addition to re-irradiation for malignant glioma	Voss et al.	Calorically-restricted ketogenic diet and intermittent fasting	Energy modified diet	32619561
Effect of glutamine-enriched nutritional support on intestinal mucosal barrier function, MMP-2, MMP-9 and immune function in patients with advanced gastric cancer during perioperative chemotherapy	Wang et al.	Patenteral nutrition with glutamine supplementation	Nutrition support	28927119
Effects of glutamine on plasma endotoxin and immune function in postperative patients with laryngeal carcinoma	Wang et al.	Parenteral nutrition containing glutamine-bipeptide	Nutrition support	Not found
Clinical trial: Efficacy of a low or modified fat diet for the prevention of gastrointestinal toxicity in patients receiving radiotherapy treatment for pelvic malignancies	Wedlake et al.	Low-fat diet	Energy modified diet	22515941
High fibre diet, low fibre diet or habitual diet for the prevention of radiation-induced toxicity in pelvic cancer: A multi-centre randomised controlled trial	Wedlake et al.	High-fiber diet	Fiber modified diet	Not found
Dietary supplementation with n-3-fatty acids in patients with pancreatic cancer and cachexia: marine phospholipids versus fish oil - a randomized controlled double-blind trial	Werner et al.	Fish oil supplementation	Nutrient modified diet	28578704
Effects of Perioperative Oral Nutrition Supplementation in Malaysian Patients Undergoing Elective Surgery for Breast and Colorectal Cancers—A Randomised Controlled Trial	Wong et al.	Perioperative ONS supplementation	Nutrient modified diet	35276977
A study of caloric restriction versus standard diet in overweight men with newly diagnosed prostate cancer: A randomized controlled trial	Wright et al.	Low calorie diet	Energy modified diet	23775525
A randomized controlled trial of postoperative artificial nutrition in malnourished patients with gastrointestinal cancer	Wu et al.	Mixed enteral/parenteral artificial nutrition	Nutrition support	18000777
Preoperative enteral immunonutrition improves postoperative outcome in patients with gastrointestinal cancer	Xu et al.	Preoperative immunonutrition	Nutrient modified diet	16830214
Effect of a nutritional therapy using a late evening snack for hepatocellular carcinoma	Yamasaki et al.	BCAA supplementation	Nutrient modified diet	Not found
Impact of Enhanced Recovery after Surgery with Preoperative Whey Protein-Infused Carbohydrate Loading and Postoperative Early Oral Feeding among Surgical Gynecologic Cancer Patients: An Open-Labelled Randomized Controlled Trial	Yi et al.	Preoperative whey protein-infused carbohydrate loading	Nutrient modified diet	31968595
Impact of perioperative enteral immunonutrition in patients with gastrointestinal cancer undergoing elective surgery: a randomized controlled trial	Yu et al.	Immunomodulating nutrition	Nutrient modified diet	Not found
Effect of Qihuang Decoction Combined with Enteral Nutrition on Postoperative Gastric Cancer of Nutrition and Immune Function	Yu et al.	Qihuang decoction supplement	Nutrient modified diet	32215032
Impact of hypocaloric and hyponitrogenic parenteral nutrition on clinical outcome in postoperative patients: A multi-center randomized controlled trial of 120 cases	Zhan et al.	Hypocaloric and hypo-nitrogen parenteral nutrition	Nutrition support	17919374
Roles of clinical pharmacists in nutritional therapy for gastric cancer patients treated with neoadjuvant chemotherapy	Zhang et al.	Nutrition counseling from clinical pharmacists	Nutrition counseling	Not found
Effect of postoperative precision nutrition therapy on postoperative recovery for advanced gastric cancer after neoadjuvant chemotherapy	Zhao et al.	Postoperative precision nutrition therapy	Nutrition support	Not found
Nutritional support of pediatric patients with cancer consuming an enteral formula with fructooligosaccharides	Zheng et al.	Fructooligosaccharide supplement	Nutrition support	Not found
Effect of oral nutritional supplementation on the post-discharge nutritional status and quality of life of gastrointestinal cancer patients after surgery: a multi-center study	Zhu et al.	Perioperative oral nutritional supplementation	Nutrient modified diet	31464391

Improvement of constipation in leukemia patients undergoing chemotherapy using sweet potato	Zou et al.	Sweet potato consumption	Nutrient modified diet	25881813
